# Blinded *In Silico* Drug Trial Reveals the Minimum Set of Ion Channels for Torsades de Pointes Risk Assessment

**DOI:** 10.3389/fphar.2019.01643

**Published:** 2020-01-30

**Authors:** Xin Zhou, Yusheng Qu, Elisa Passini, Alfonso Bueno-Orovio, Yang Liu, Hugo M. Vargas, Blanca Rodriguez

**Affiliations:** ^1^ Department of Computer Science, British Heart Foundation Centre of Research Excellence, University of Oxford, Oxford, United Kingdom; ^2^ SPARC, Amgen Research, Amgen Inc., Thousand Oaks, CA, United States; ^3^ GAU, Amgen Research, Amgen Inc., South San Francisco, CA, United States

**Keywords:** Torsades de Pointes, drug cardiotoxicity, ion channels, *in silico* drug trials, human ventricular action potential

## Abstract

Torsades de Pointes (TdP) is a type of ventricular arrhythmia which could be observed as an unwanted drug-induced cardiac side effect, and it is associated with repolarization abnormalities in single cells. The pharmacological evaluations of TdP risk in previous years mainly focused on the hERG channel due to its vital role in the repolarization of cardiomyocytes. However, only considering drug effects on hERG led to false positive predictions since the drug action on other ion channels can also have crucial regulatory effects on repolarization. To address the limitation of only evaluating hERG, the Comprehensive in Vitro Proarrhythmia Assay initiative has proposed to systematically integrate drug effects on multiple ion channels into *in silico* drug trial to improve TdP risk assessment. It is not clear how many ion channels are sufficient for reliable TdP risk predictions, and whether differences in IC_50_ and Hill coefficient values from independent sources can lead to divergent *in silico* prediction outcomes. The rationale of this work is to investigate the above two questions using a computationally efficient population of human ventricular cells optimized to favor repolarization abnormality. Our blinded results based on two independent data sources confirm that simulations with the optimized population of human ventricular cell models enable efficient in silico drug screening, and also provide direct observation and mechanistic analysis of repolarization abnormality. Our results show that 1) the minimum set of ion channels required for reliable TdP risk predictions are Nav1.5 (peak), Cav1.2, and hERG; 2) for drugs with multiple ion channel blockage effects, moderate IC_50_ variations combined with variable Hill coefficients can affect the accuracy of *in silico* predictions.

## Introduction

Cardiotoxicity is a major cause of drug withdrawal from the pharmaceutical market, and its earlier detection and assessment could largely speed up the evaluation of target compounds in the drug development process. For instance, drug-induced Torsades de Pointes (TdP) is a type of ventricular arrhythmia linked to sudden cardiac death. It is generally accepted that early afterdepolarizations (EADs) occurring during the repolarization phase of action potentials (APs) can trigger premature events, and then give rise to TdP (Vandersickel et al., 2014: Vandersickel et al., 2015). Drugs that block the hERG current (I_Kr_) can inhibit the repolarization process, leading to AP duration (APD) prolongation, and facilitating EAD generation (Jurkiewicz and Sanguinetti, 1993; Guo et al., 2011; Pueyo et al., 2011; Dutta et al., 2016). Therefore, prolongation of APD (reflected in the electrocardiogram as QTc interval prolongation) is often used as a surrogate to assess TdP risk. I_Kr_ inhibition and QTc prolongation are sensitive but not very specific for predicting ventricular pro-arrhythmia risk. Inhibition of other cardiac ion channels, especially sodium and calcium channels, may mitigate the effects of hERG blockage and reduce pro-arrhythmic risk and EAD generation (Bril et al., 1996; Martin et al., 2004; Sager et al., 2014; Britton et al., 2017).

Since hERG assays alone can lead to false positives in predicting TdP risk and repolarization abnormalities, Kramer et al. measured concentration-response relationships of hERG, Nav1.5 peak (the fast sodium current, I_Na_), and Cav1.2 (the L-type calcium current, I_CaL_) currents for 32 torsadogenic and 23 non-torsadogenic drugs from multiple pharmacological classes (Kramer et al., 2013). A logistic regression analysis showed that risk prediction based on the three channels improved accuracy with respect to using solely hERG block (Kramer et al., 2013).

To overcome the limitation of hERG inhibition as the main evaluation criteria, the Comprehensive in Vitro Proarrhythmia Assay (CiPA) initiative, sponsored by the Food and Drug Administration (FDA) among others, has proposed a new paradigm to integrate drug effects on multiple ion channels into *in silico* modeling to evaluate the propensity for EADs and repolarization instabilities (Sager et al., 2014; Colatsky et al., 2016). Driven by the scheme of CiPA, recent experimental studies have comprehensively analyzed the effects of clinical drugs on up to seven ionic currents (Crumb et al., 2016).

Building on *in vitro* ion channel data, *in silico* TdP risk prediction has shown promising results using several approaches. One strategy for *in silico* TdP risk stratification is to utilize machine learning algorithms to derive TdP risk metrics. Strong performance was achieved using principal component analysis, support vector machine classifications, and logistic regression classifiers, based on ion channel blockage data and features of simulated AP and intracellular calcium transients (Lancaster and Sobie, 2016; Parikh et al., 2017). Another strategy is to use simple classification models to analyze the balance between depolarization and repolarization blockages (Mistry et al., 2015). However, statistical analysis and machine learning classifications do not enable direct observations of pro-arrhythmic repolarization abnormalities (RAs), and make mechanistic interpretations difficult. Alternatively, using RAs as the main metric, simulations using a population of over 1,000 human ventricular cell models with random ionic current variations yielded 89% accuracy in TdP predictions (Passini et al., 2017). The accuracy was higher using RAs in the virtual human cell population than using a single model or APD prolongation, and also higher compared to experimental animal models. The simulation results also revealed mechanistic ion channel properties underlying RAs: for example, weaker I_NaK_ (the sodium potassium pump current) favored RA, which was related to proarrhythmic clinical conditions such as acute myocardial ischemia (Passini et al., 2017).

Normal repolarization involves complex interactions and contributions of multiple ionic currents, which include some redundancy for the robustness of this critical part of the cardiac cycle, namely the so-called repolarization reserve (Roden, 1998; Roden Dan, 2008; Roden and Abraham, 2011). The subclinical change in some currents may not directly lead to RA but the lower repolarization reserve in these scenarios provides vulnerable conditions for RA generation when ion channel blockers are superimposed (Roden, 1998; Roden Dan, 2008; Roden and Abraham, 2011). Inspired by the mechanistic ion channel properties revealed in a previous study (Passini et al., 2017), a population of human ventricular cell models was designed to favor RA by introducing lower repolarization reserve as weaker repolarization currents and stronger depolarization currents (Passini et al., 2019).

In this study, we performed blinded *in silico* drug trials for 85 reference compounds using the optimized virtual human cell population by (Passini et al., 2019), to investigate the following questions: 1) what is the minimum set of ion channels needed for good TdP risk predictions; 2) how different are *in silico* prediction outcomes using IC_50_ and Hill coefficient values from two independent and highly cited sources?

The significance of blinding in this study is twofold: 1) no iterations of model calibration were done to improve predictions, and this enabled a clear and independent validation of the simulations for the use of drug induced TdP risk prediction; 2) blinding also mimicked the preclinical stage of drug development when the side effects were unknown before large clinical trials, and no additional information such as the effects of metabolites can be taken into account, therefore the blinded simulations reflected better the new compound evaluation process. The accuracy of the blinded prediction and its low computational cost demonstrated the potential applicability of this *in silico* approach in cost and animal use reduction for drug development in pharmaceutical industry.

## Materials and Methods

### Optimization of the Population of Human Ventricular Models

The baseline model used in this study is the O’Hara-Rudy dynamic (ORd) human endocardial ventricular AP model (O’Hara et al., 2011), developed using data from over 100 human hearts, with modifications to the fast sodium channel (Passini et al., 2016). It includes detailed representation of the potassium, sodium and calcium sarcolemmal currents, as well as intracellular calcium handling (including SERCA pump and calcium-induced calcium release mechanism). A computationally efficient population of 107 human ventricular AP models constructed in (Passini et al., 2019) was used for the assessment of RAs, as a surrogate for pro-arrhythmic mechanisms under drug action. Based on established knowledge of ionic profiles more likely to develop drug induced RA (Passini et al., 2017), the population of models was constructed by varying nine key ionic conductances to represent electrophysiological variability, then constrained and calibrated using the human data (Britton et al., 2017). Conductances were varied unevenly to allow weaker I_Kr_, I_Ks_ (the slow delayed rectifier potassium current) and I_NaK_, and stronger I_NaL_ (the late sodium current), I_CaL,_ and I_NaCa_ (the sodium-calcium exchanger current) (**Supplementary Material Table S1**) (Passini et al., 2019). The low repolarization reserve introduced in the population is also representative of different pathological conditions, such as long QT syndrome (Schwartz Peter et al., 2012), hypertrophic cardiomyopathy (Coppini et al., 2013), and heart failure (Shamraj et al., 1993; Ambrosi et al., 2013). The full list of parameters for the optimized population is provided in the **Supplementary Material Table S5**.

### Datasets

The sources and the names of all the compounds were blinded during the *in silico* test. *In silico* drug trials were performed for two groups of reference compounds at multiple concentrations. Drug/ionic current interactions were simulated using a simple pore-block drug model (Brennan et al., 2009). For each drug, IC_50_ and Hill coefficient were the input parameters used to describe each ionic current block as in previous studies (Mirams et al., 2011; Passini et al., 2017).

Dataset I consisted of 30 compounds, for which IC_50_ and Hill coefficients were available for seven ionic currents: I_Na_, I_NaL_, I_to_ (the transient outward potassium current), I_Kr_, I_Ks_, I_K1_ (the inward rectifier potassium current), and I_CaL_ (Crumb et al., 2016). Dataset II had 55 compounds, with IC_50_ and Hill coefficients available for only three ion channels: I_Na_, I_Kr_, and I_CaL_ (Kramer et al., 2013). The full lists of IC_50_ and Hill coefficient values for both datesets are provided in the **Supplementary Material Table S6**. For each compound, five concentrations were tested: 1, 3, 10, 30, and 100 folds of their maximal effective free therapeutic concentration (EFTPC_max_).

### Simulation Environment

Virtual Assay (v.2.4.800 © 2014 Oxford University Innovation Ltd. Oxford, UK), a user-friendly C++ based software package was previously developed for the simulation of drug effects on virtual cardiac AP populations. Virtual Assay uses the ordinary differential equation solver CVODE to implement backward differentiation formula with adaptive time steps (Hindmarsh et al., 2005; Serban and Hindmarsh, 2008), and relative and absolute tolerances equal to 1e-5 and 1e-7, respectively. As shown previously in (Passini et al., 2017), similar simulations of virtual AP traces can be achieved using MATLAB (Mathworks Inc. Natwick, MA, USA) or any other software to solve ordinary differential equations. The effect of a drug at a testing concentration was simulated as conductance reductions which were based on the IC_50_ values and Hill coefficients from the blinded datasets. For each concentration of each compound, the drug assay was conducted on the population of models at a pacing frequency of 1Hz for 150 beats, and the AP traces of the last beat were used for analysis as in (Passini et al., 2017). The choice of the pacing frequency was based on clinical reports that pacing rates slower than 70 beats per minute were more relevant to TdP occurrence while faster pacing can suppress TdP (Viskin et al., 2000; Pinski et al., 2002; Kurisu et al., 2008). For all the simulated traces, APD was calculated as APD_90_ (AP duration at 90% of repolarization), and TdP risk prediction was conducted by analyzing the morphology of simulated AP traces to detect RAs, as described below.

### *In silico* TdP Risk Analysis

TdP risk prediction was calculated based on the occurrence of two types of RAs in the population of human models, encompassing EADs and repolarization failure (RF). The two types of RAs were defined when positive derivatives were found in the membrane voltage (V_m_) after 150 ms following the AP peak (for EADs), or the membrane voltage failed to reach resting membrane voltage (V_m_ >-40 mV) at the end of the last beat (for RF). In order to ensure the accuracy of automated RA detection, simulation traces were also visually examined to check for potential misclassifications caused by the algorithm.

Aggregated results at the population level are presented using a scoring system (TdP risk score) as introduced in (Passini et al., 2017) according to the following formula (where nRA*c* is the number of models showing RA at a tested concentration *c*, W*c* = EFTPC_max_ / *c* is the weight inversely related to the tested concentration *c*, and N is the total number of models in the population).

$$\mathrm{TdP}\;\mathrm{risk}\;\mathrm{score}=\frac{\sum_{c}\left(\text{Wc}*\text{nRAc}\right)}{\text{N}*\sum_{c}\text{Wc}}$$

The analysis of TdP scores was also performed blindly. The TdP risk score integrated RA occurrence at multiple concentrations, and was computed in MATLAB. Logarithmic scale was considered to maximize the visual separation of TdP score between safe and risky drugs. Zero risk was approximated for visualization with machine precision (10^−16^). For special cases where access to individual ionic currents and ion channel gating variables was needed, the same drug trials were repeated in MATLAB to obtain more detailed information.

### Evaluations of the *In Silico* Prediction Results

After the blinded *in silico* prediction, the TdP risk of each compound was compared against the current clinical reports in the literature. Multiple sources were referenced for classification as in (Kramer et al., 2013), including CredibleMeds^®^ (Woosley et al, 2019), available on www.crediblemeds.org, (Redfern et al., 2003), publications on QT studies, and FDA-generated package inserts. Considering the risk classifications of some drugs are debatable, we also evaluated the prediction accuracy by only applying the CredibleMeds^®^ classification. For this, compounds with any type of TdP risk (known, possible or conditional) were considered to be risky even if the risk may only occur under specific conditions such as overdose or interactions with other drugs (**Supplementary Material** **Figures S1–S4**, **Tables S2–S4**).

Prediction results were classified as true positive (TP, both clinical reports and *in silico* predictions were risky), true negative (TN, both clinical reports and *in silico* predictions were safe), false positive (FP, no clinical risk reports but *in silico* predictions were risky), and false negative (FN, *in silico* predicted to be safe but clinically reported to be risky). Sensitivity, specificity, positive predictive value (PPV), and negative predictive value (NPV) were computed separately for Dataset I and II based on the number of TP, TN, FP, and FN predictions. Accuracy was defined as the sum of TPs and TNs divided by the total number of drugs. Each drug is referred to with the name and a roman number to differentiate the two datasets.

### Statistical Analysis

The formula for TdP score calculation is a deterministic algorithm using the same models for all drugs, and therefore no statistical algorithm was used for calculating TdP risk for individual drugs. Pairwise linear correlation was performed between TdP scores and drug induced APD prolongations using MATLAB.

## Results

### *In Silico* Prediction of TdP Risk Considering All Available Ion Channel Data and All Assessed Concentrations

**Figure 1:**
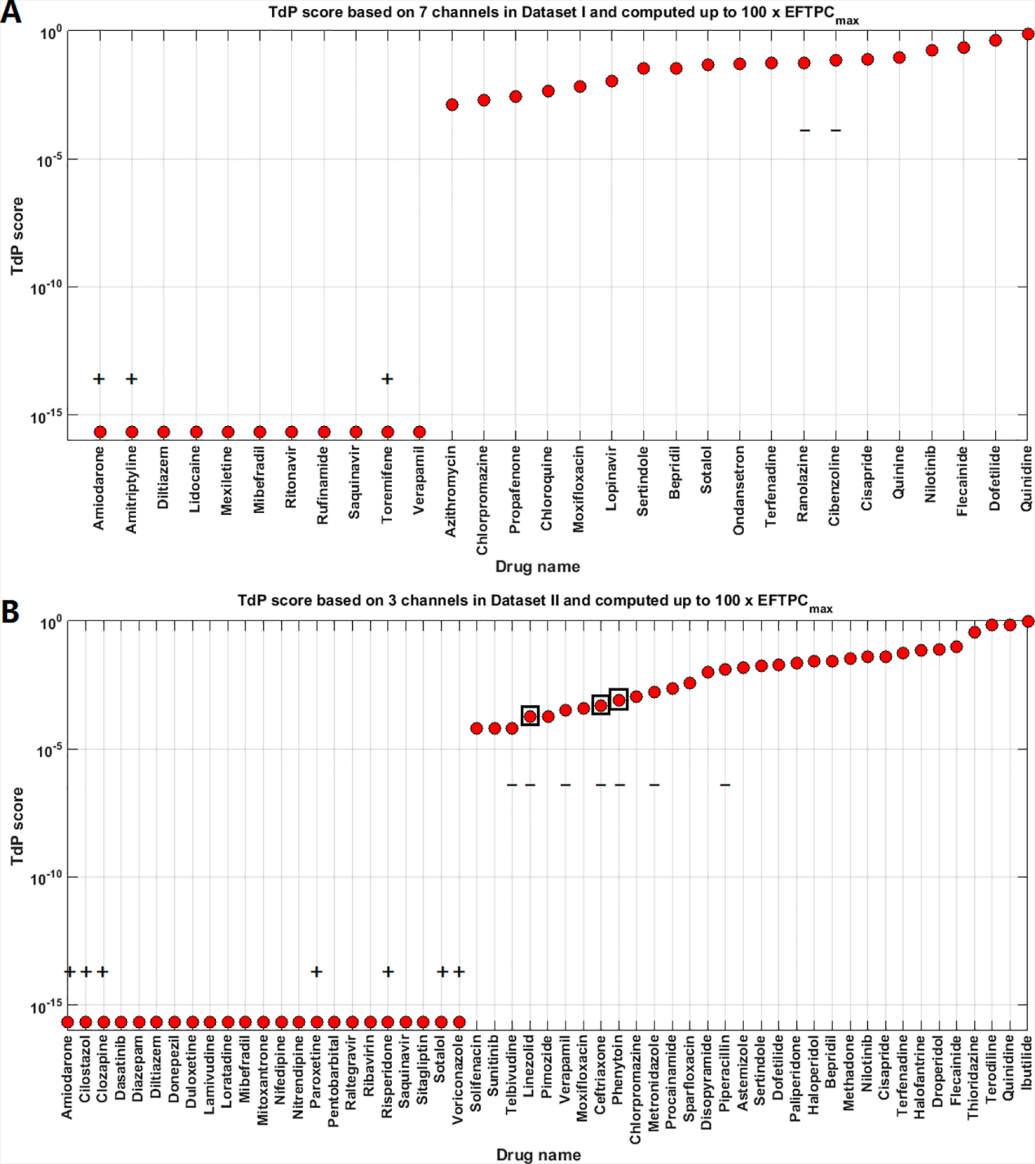
Torsades de Pointes (TdP) risk assessment based on all the available ion channel inputs and up to 100×EFTPC_max_. **(A)** Dataset I TdP risk assessment based on all data from 7 ion channels (Crumb et al., 2016). **(B)** Dataset II TdP risk assessment based on all information from 3 ion channels (Kramer et al., 2013). Classifications inconsistent with current clinical reports are labeled with black “+” and “–”: “+” implies the true classification should be risky, while “–” means the true classification should be safe. Black squares in panel **(B)** highlight the compounds whose TdP risk was generated by the misclassification of the automated algorithm, and visual examinations of the simulated AP traces revealed they did not produce repolarization abnormalities (RAs).

The TdP risk scores were computed for both groups of compounds, and in both cases risky and safe compounds were identified. In Dataset I, unblinding the compounds revealed a sensitivity of 85% and a specificity of 80% (**Table 1**), and the overall accuracy was 83%, with 3 FN predictions and 2 FP predictions. As illustrated in **Figure 1A** for Dataset I, simulations with 11 out of 30 compounds yielded no RAs and thus no TdP risk, whereas 19 compounds induced RA in the population of 107 human ventricular cell models. Among the Dataset I compounds that produced RAs, Quinidine I demonstrated the highest risk, with a TdP score of 0.78, while Azithromycin I showed a mild risk with a TdP score of 10^-3^.

**Table 1:** Accuracy of the *in silico* Torsades de Pointes (TdP) risk predictions using the population of 107 human ventricular cell models with maximum testing concentrations of 100×EFTPC_max_.

	Dataset I		Dataset II		
	Clinical TdP (+)	Clinical TdP (-)	Clinical TdP (+)	Clinical TdP(-)	
*In silico* (+)	17	2	25	7	**4**
*In silico* (-)	3	8	7	16	**19**
Sensitivity	85%		78%	**78%**	
Specificity	80%		70%	**83%**	
PPV	89%		78%	**86%**	
NPV	73%		70%	**73%**	
Accuracy	83%		75%	**80%**	

For Dataset II, bold scores indicate the true accuracy of the model predictions after fixing the misclassifications of the automated repolarization abnormality (RA) detection algorithm.

In Dataset II, 23 out of 55 compounds did not induce RAs, while the remaining 22 compounds produced RAs according to the automated RA detection algorithm (**Figure 1B**). Ibutilide II had the highest TdP score of 1, and Solifenacin II had a very mild TdP score of 6·10^-5^. Unblinding the compounds revealed a sensitivity of 78% and a specificity of 70% (**Table 1**), with 7 FN predictions and 7 FP predictions from the automated RA detection.

Further visual examination of the raw AP traces revealed that 3 of the risky compounds detected by the automated algorithm (highlighted in black squares in **Figure 1B**) did not induce RAs, and the misclassification was caused by delayed AP peaks (**Supplementary Material**, **Figures S5A–C**). The misclassifications only happened for traces produced under 100×EFTPC_max_. Fixing the misclassification caused by the automated RA detection algorithm revealed simulations had a true sensitivity of 78%, a specificity of 83%, and an overall accuracy of 80% (**Table 1**, highlighted in bold).

The overall prediction accuracy against the CredibleMeds^®^ drug classifications was similar (**Supplementary Material Figure S1**, **Table S2**). There were however some differences. Our classifications of Saquinavir, Ranolazine, Dasatinib, Donepezil, Metronidazole, Piperacillin were non-risky based on multiple sources such as CredibleMeds^®^, publications on QT studies, and package labels, but the latest CredibleMeds^®^ database (accessed 2018-11-28) classify them as risky. The simulation results showed Ranolazine, Metronidazole, and Piperacillin induced RA, while Saquinavir, Dasatinib, and Donepezil did not induce RA.

After unblinding the compounds, we explored whether different pacing rates can affect the predictive accuracy of the simulations by applying a faster pacing rate of 2Hz and a slower pacing rate of 0.5 Hz at 10x EFTPC_max_ for the compounds in Dataset I. The simulation results showed that slower pacing tended to induce more EADs (**Supplementary Figure S7**), but no qualitative differences were observed for the FN (Amiodarone I, Amitriptyline I and Toremifene I) and FP compounds in terms of RA generation (Cibenzoline I and Ranolazine I) (**Supplementary Figures S8** and **S9**).

### Action Potential Prolongation and TdP Risk

**Figure 2:**
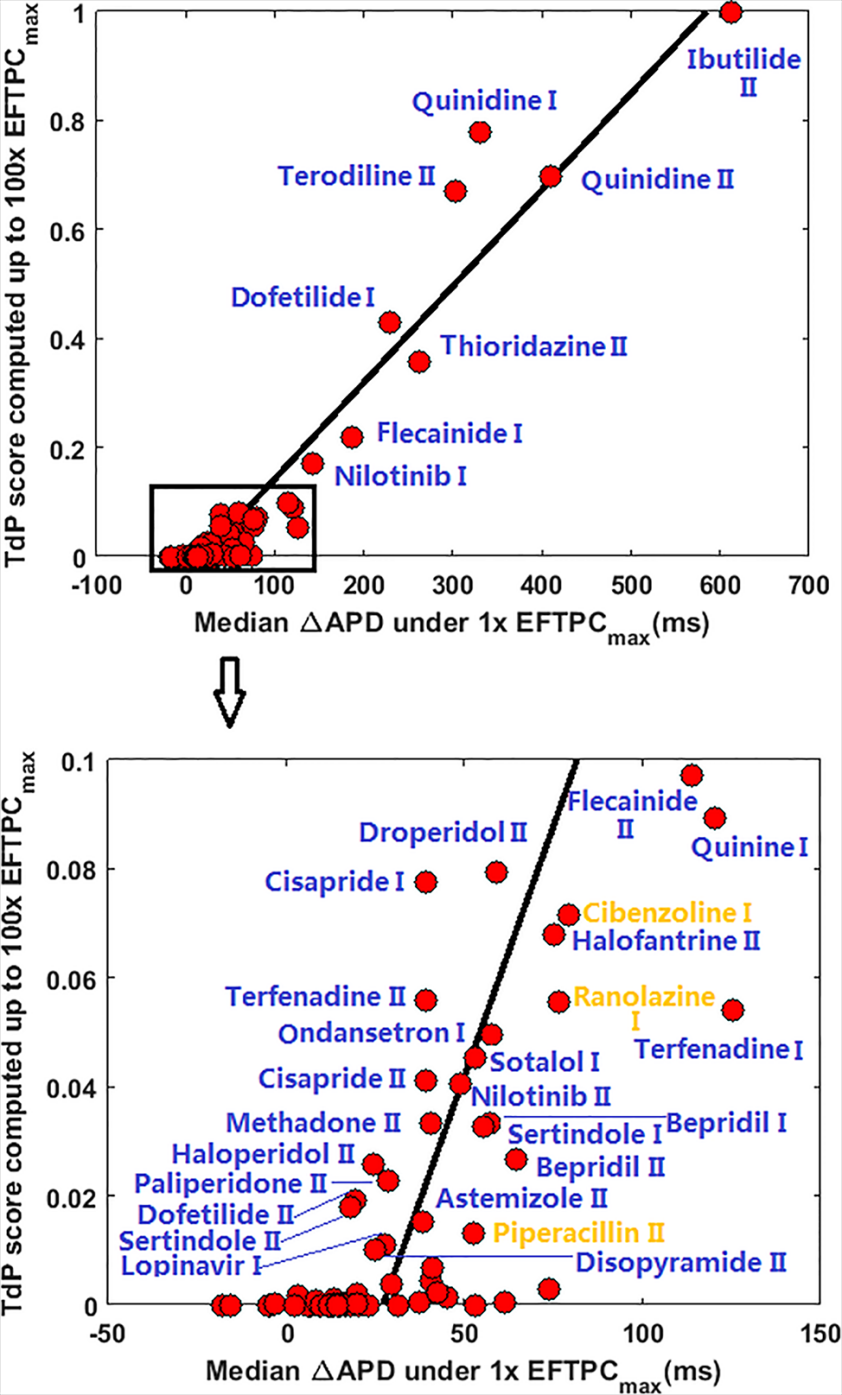
Correlation between action potential duration (APD) prolongation and Torsades de Pointes (TdP) risk score at 1×EFTPCmax for all 85 compounds. ΔAPD=APDdrug-APDcontrol. Blue labels indicate true positive predictions, and yellow labels indicate false positive results. If CredibleMeds® classification was used, Ranolazine I and Piperacillin II would be true positive (blue) instead of false positive.

**Figure 3:**
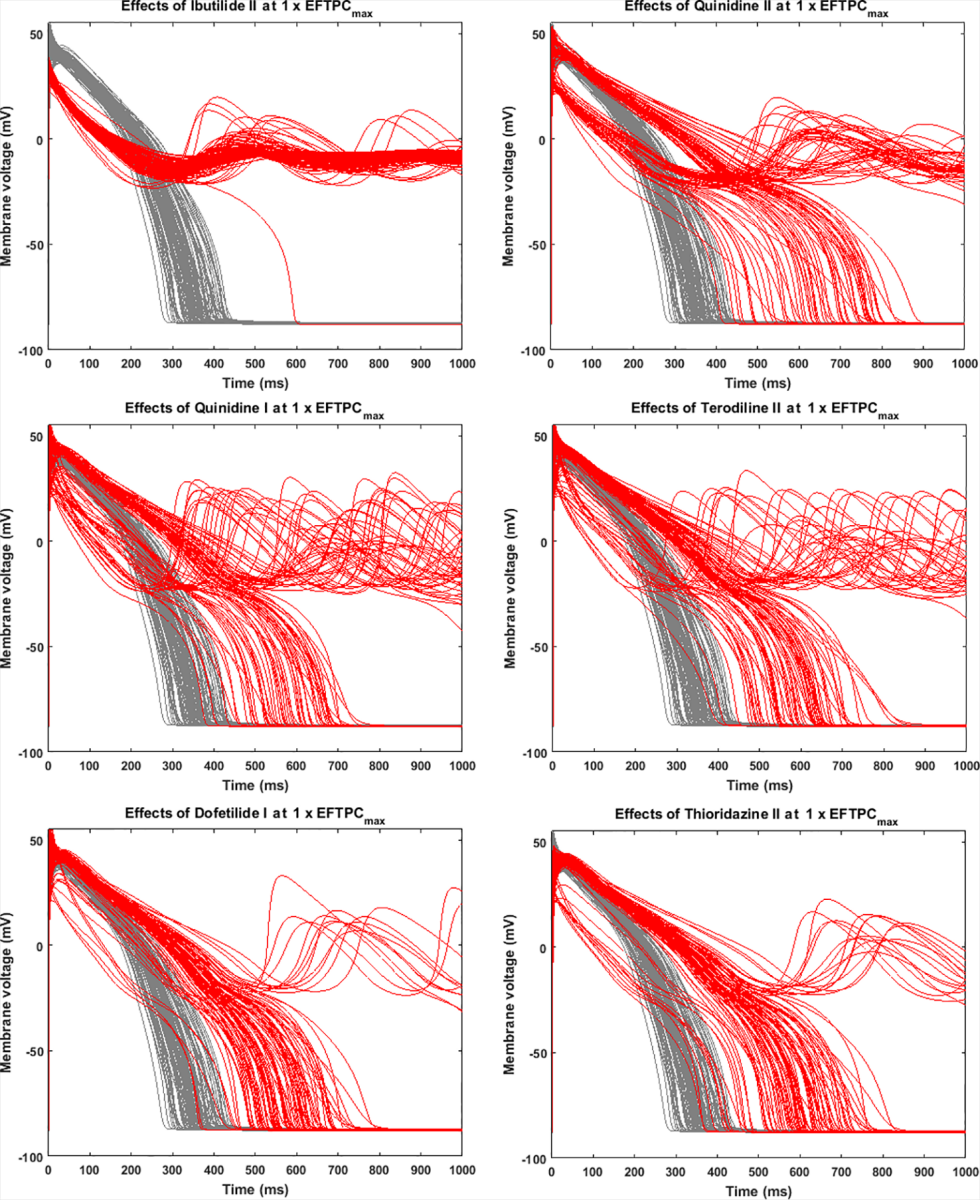
Representative compounds that induce repolarization abnormalities at 1×EFTPC_max_. Grey, control condition; red, drug action.

**Figure 4:**
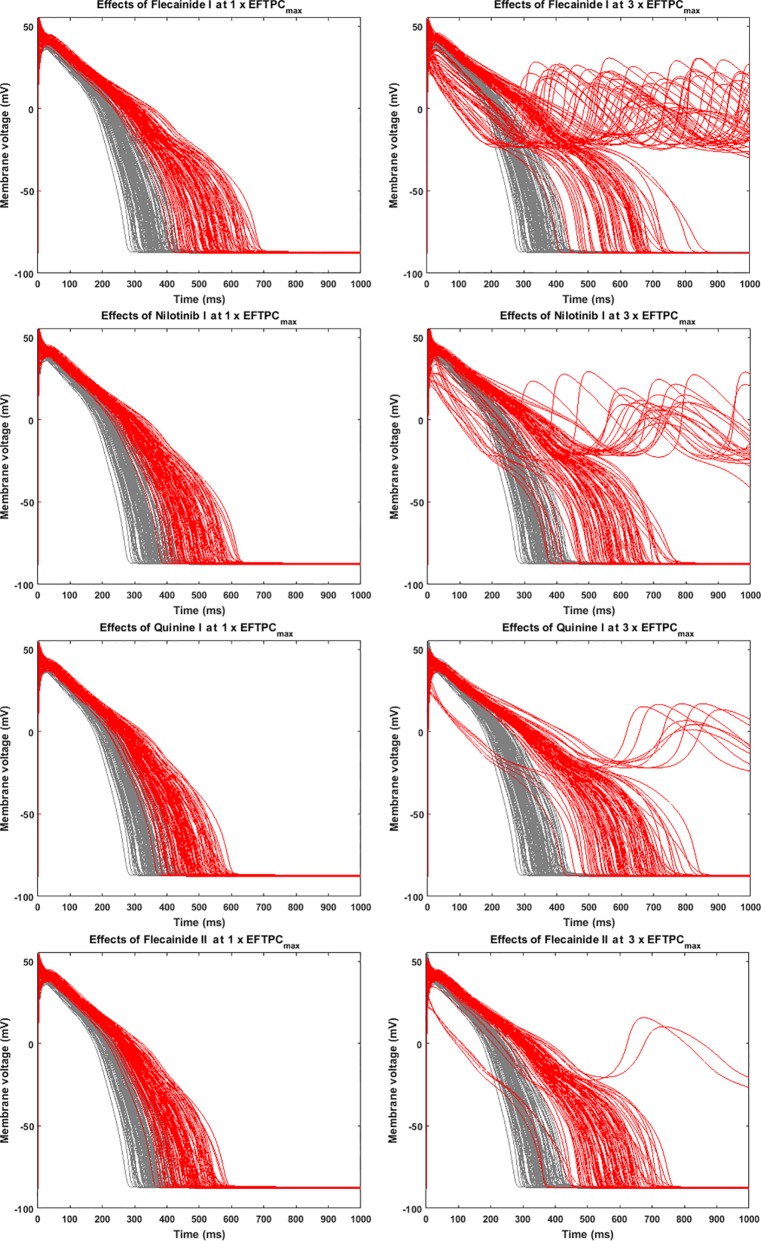
Representative compounds that prolong action potential duration (APD) at 1×EFTPC_max_ and induce repolarization abnormalities at 3×EFTPC_max_. Grey, control condition; red, drug action.

Since many compounds can block hERG and cause prolongation of APD, and excessive APD prolongation can also be a TdP risk factor, the relationship between TdP risk and APD prolongation at low doses was also evaluated. A good linear correlation was found between the overall TdP risk score and the APD prolongation at 1 × EFTPC_max_ (r^2^ = 0.96) (**Figure 2**, upper panel). Ibutilide II, which had the biggest TdP score of 1 and yielded the biggest APD prolongation, induced RFs in the population even at the lowest tested dose. Quinidine I (TdP score = 0.78), Quinidine II (TdP score = 0.70), Terodiline II (TdP score = 0.67), Dofetilide I (TdP score = 0.43), and Thioridazine II (TdP score = 0.36) induced significant APD prolongation and EADs at the lowest testing dose (**Figure 3**). Flecainide I (TdP score = 0.22), Nilotinib I (TdP score = 0.17), Quinine I (TdP score = 0.09), Terfenadine I (TdP score = 0.05), and Flecainide II (TdP score = 0.10) did not induce RAs at 1×EFTPC_max_. However, they can lead to APD prolongation of more than 100 ms, and four of these compounds led to EADs when the testing concentration was increased to 3×EFTPC_max_ (**Figure 4**).

Although a good linear correlation was found between very high risk TdP scores and APD prolongation at 1×EFTPC_max_, APD prolongation was less well correlated with low TdP risk scores. As shown in the lower panel of **Figure 2**, with similar low TdP scores close to 0, some compounds showed APD prolongations of up to 70 ms, while others showed very small APD prolongations of less than 20 ms.

### Reducing Maximum Testing Concentration Does Not Improve the RA-Based TdP Risk Stratification

**Figure 5:**
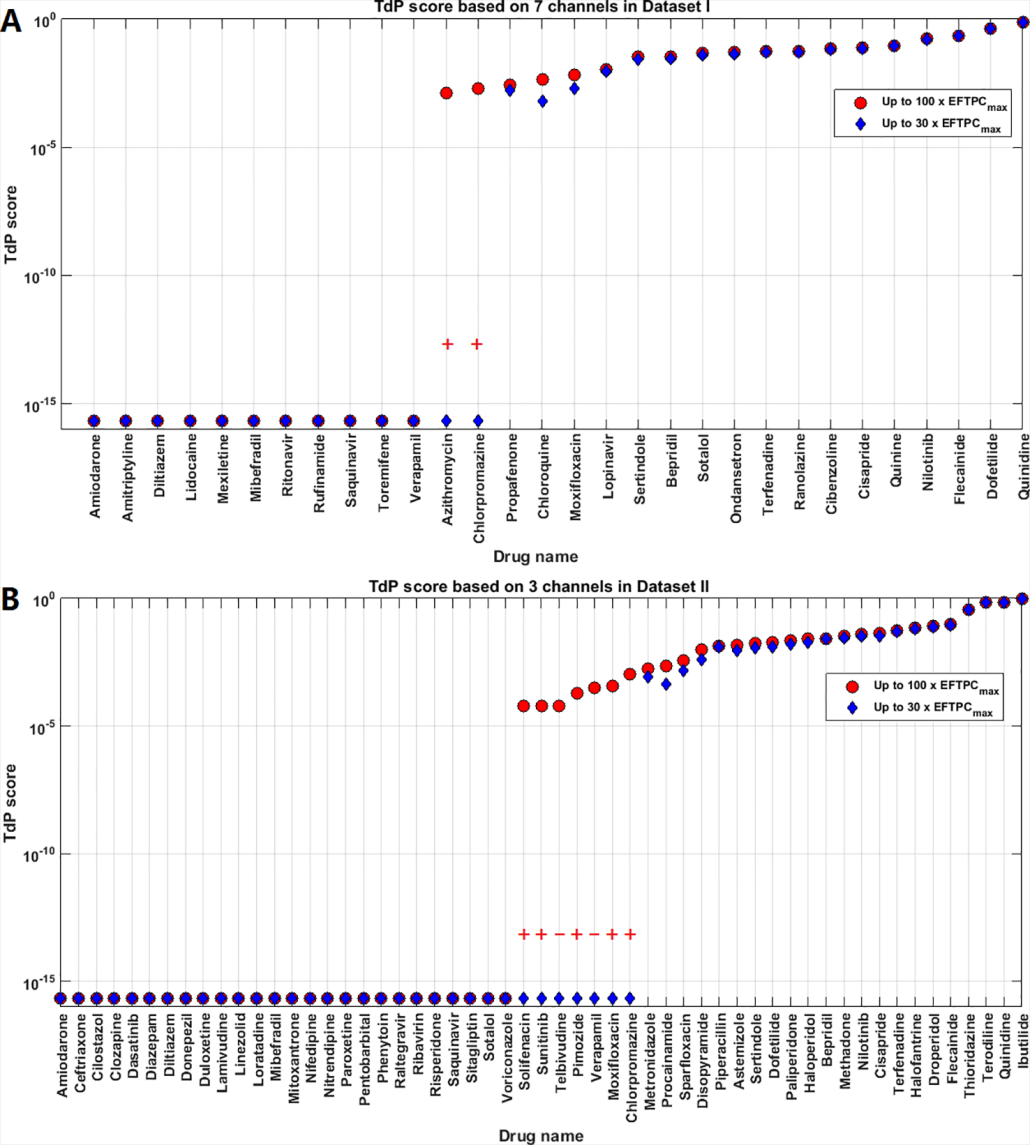
Comparison of Torsades de Pointes (TdP) risk between up to 30×EFTPC_max_ and 100×EFTPC_max_ for Dataset I **(A)** and Dataset II **(B)**. Red “+” and “–” show the classification changes caused by lower testing concentrations: “+” implies the true classification should be risky, while “–” means the true classification should be safe. The three Dataset II compounds whose TdP risk was misclassified by the automated algorithm were corrected in this figure.

In the clinical situation, the maximally-achieved plasma exposure is determined by drug absorption, distribution, metabolism and excretion in an individual whose genetic background and physical condition have significant effects on pharmacokinetics (Tamargo et al., 2017). Therefore, we considered high concentrations of up to 100×EFTPC_max_ in the formerly discussed evaluation of TdP risk. Some compounds with intermediate TdP risk scores elicited RAs only at the highest tested concentration. In order to assess the effects of maximum tested concentrations, we also compared the TdP score calculated with maximum concentrations up to 30×EFTPC_max_. As shown in **Figure 5A** (Dataset I) and **Figure 5B** (Dataset II), predictions were not significantly affected for drugs with either the highest or lowest TdP scores. However, in a few cases, the risk classification can be qualitatively different. For Dataset I, decreasing the maximum testing concentration to 30×EFTPC_max_ led to 2 extra FNs: Azithromycin and Chlorpromazine. Even when classified as risky at 100×EFTPC_max_, these two compounds had the lowest TdP scores among the risky category. For Dataset II, decreasing maximum concentration converted several FPs predictions to TNs but also generated 5 extra FNs.

For both groups, FN predictions cannot be improved under lower concentrations. Therefore decreasing the maximum testing concentrations reduced the sensitivity in the predictions for both datasets, while the specificity was not affected for Dataset I but was improved for Dataset II. Similar results were obtained using the latest CredibleMeds^®^ TdP risk classifications **Supplementary Material** (**Supplementary Material Figure S2**, **Table S3**). Therefore, based on the overall accuracy, changing maximum testing concentration to 30×EFTPC_max_ does not improve the quality of predictions (**Table 2**).

**Table 2:** Comparisons of the *in silico* Torsades de Pointes (TdP) risk predictions between maximum testing concentrations of 30× and 100×EFTPC_max_.

	Dataset I		Dataset II		
	100×EFTPC_max_	30×EFTPC_max_	100×EFTPC_max_		30×EFTPC_max_
Sensitivity	85%	75%	78%	**78%**	63%
Specificity	80%	80%	70%	**83%**	91%
PPV	89%	88%	78%	**86%**	91%
NPV	73%	62%	70%	**73%**	64%
Accuracy	83%	77%	75%	**80%**	75%

For Dataset II, bold scores indicate the true accuracy of the model predictions after fixing the misclassifications of the automated repolarization abnormality (RA) detection algorithm.

### Using Only hERG Decreases the Specificity of Predictions

**Figure 6:**
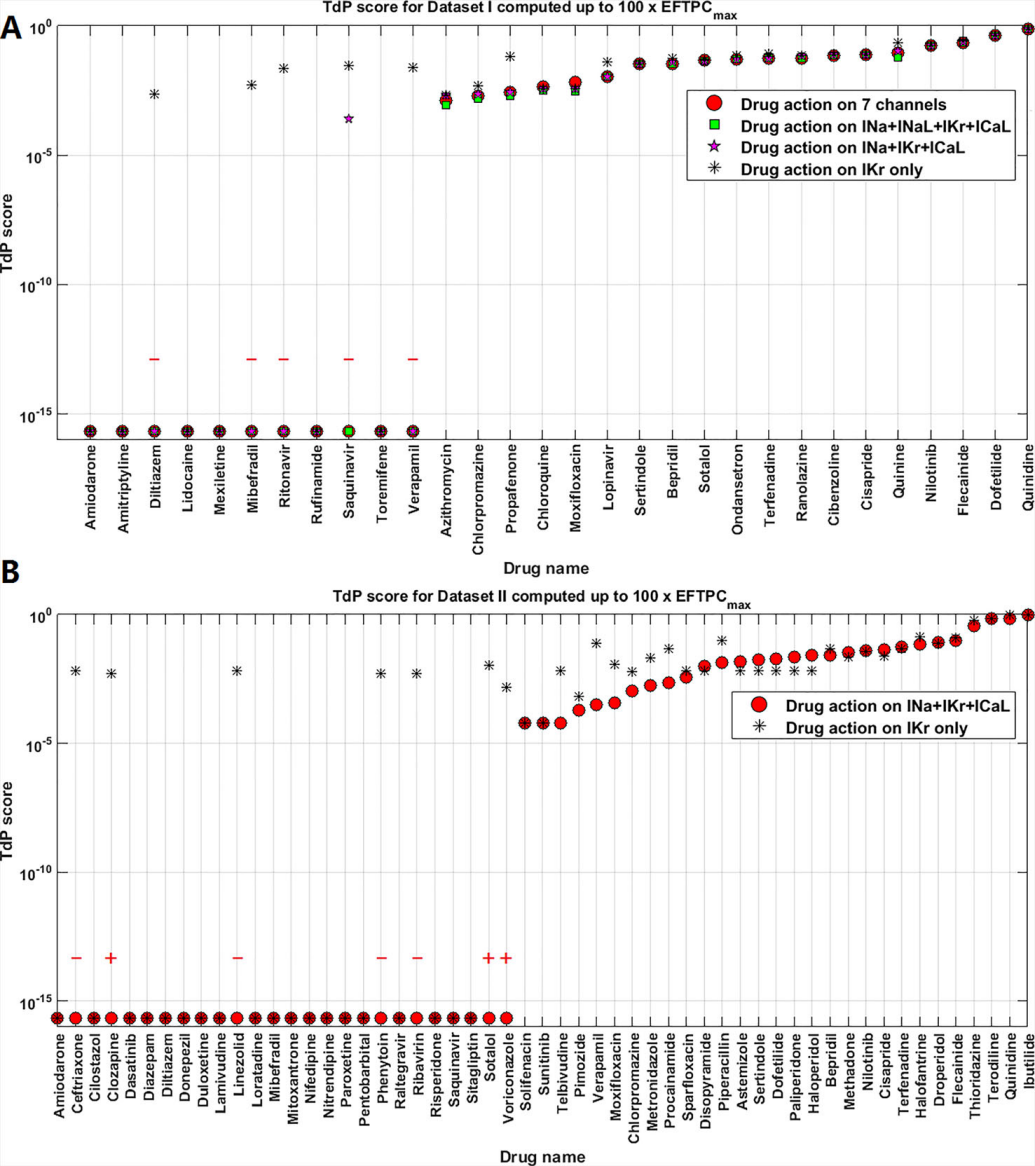
Comparison of Torsades de Pointes (TdP) risk for different subsets of ion channel information for Dataset I **(A)** and Dataset II **(B)**. Red “+” and “–” show the classification changes caused by different ion channel profiles: “+” implies the true classification should be risky, while “–” means the true classification should be safe. The three Dataset II compounds whose TdP risk was misclassified by the automated algorithm were corrected in this figure, and they did induce repolarization abnormalities (RAs) under hERG only simulations.

For Dataset I compounds, IC_50_/Hill coefficient values are available for seven ion channels, while for Dataset II the information is available for three ion channels. In order to understand how many ion channels are necessary to achieve sufficient prediction accuracy, we also compared the effect of varying the number of affected ion channels. **Figure 6A** compares prediction results for 7, 4 (I_Na_+I_NaL_+I_Kr_+I_CaL_), 3 (I_Na_+I_Kr_+I_CaL_), and 1 (I_Kr_) channel only for Dataset I. For these compounds, there was no qualitative difference between the predictions based on information from 7, 4, and 3 channels (1 exception for Saquinavir I). However, if only hERG block (I_Kr_) was considered, TdP scores were frequently higher, and five safe compounds were misclassified to FP, leading to a significant loss of specificity and overall lower accuracy (**Table 3**).

**Table 3:** Comparisons of the *in silico* Torsades de Pointes (TdP) risk predictions between different sets of ion channel profiles.

	Dataset I				Dataset II		
	7 channels	4 channels	3 channels	1 channel (hERG)	3 channels		1 channel (hERG)
Sensitivity	85%	85%	85%	85%	78%	**78%**	88%
Specificity	80%	80%	70%	30%	70%	**83%**	65%
PPV	89%	89%	85%	71%	78%	**86%**	78%
NPV	73%	73%	70%	50%	70%	**73%**	79%
Accuracy	83%	83%	80%	67%	75%	**80%**	78%

For Dataset II, bold scores indicate the true accuracy of the model predictions of 3 channels after fixing the misclassifications of the automated repolarization abnormality (RA) detection algorithm.

For Dataset II, the results from 3 channels and only hERG were also compared. Similarly, only considering I_Kr_ block increased the TdP scores in a number of cases (altering the magnitude of predicted TdP risks), and the classification of seven compounds was changed to risky (4 FP and 3 TPs, **Figure 6B**). It was noted that the three compounds misclassified by the automated algorithm when using 3 channels (Ceftriazone II, Linezolid II and Phenytoin II) did induce RAs when only considering hERG block (**Supplementary Material**, **Figures S5D–F**). Therefore, the specificity for Dataset II was also compromised as in Dataset I by using hERG only, leading to lower overall accuracy. Similar effects were observed using CredibleMeds^®^ classifications (**Supplementary Material Figure S3**, **Table S4**). Overall, considering hERG alone decreases the specificity of the predictions for both datasets (**Table 3**).

**Figure 7:**
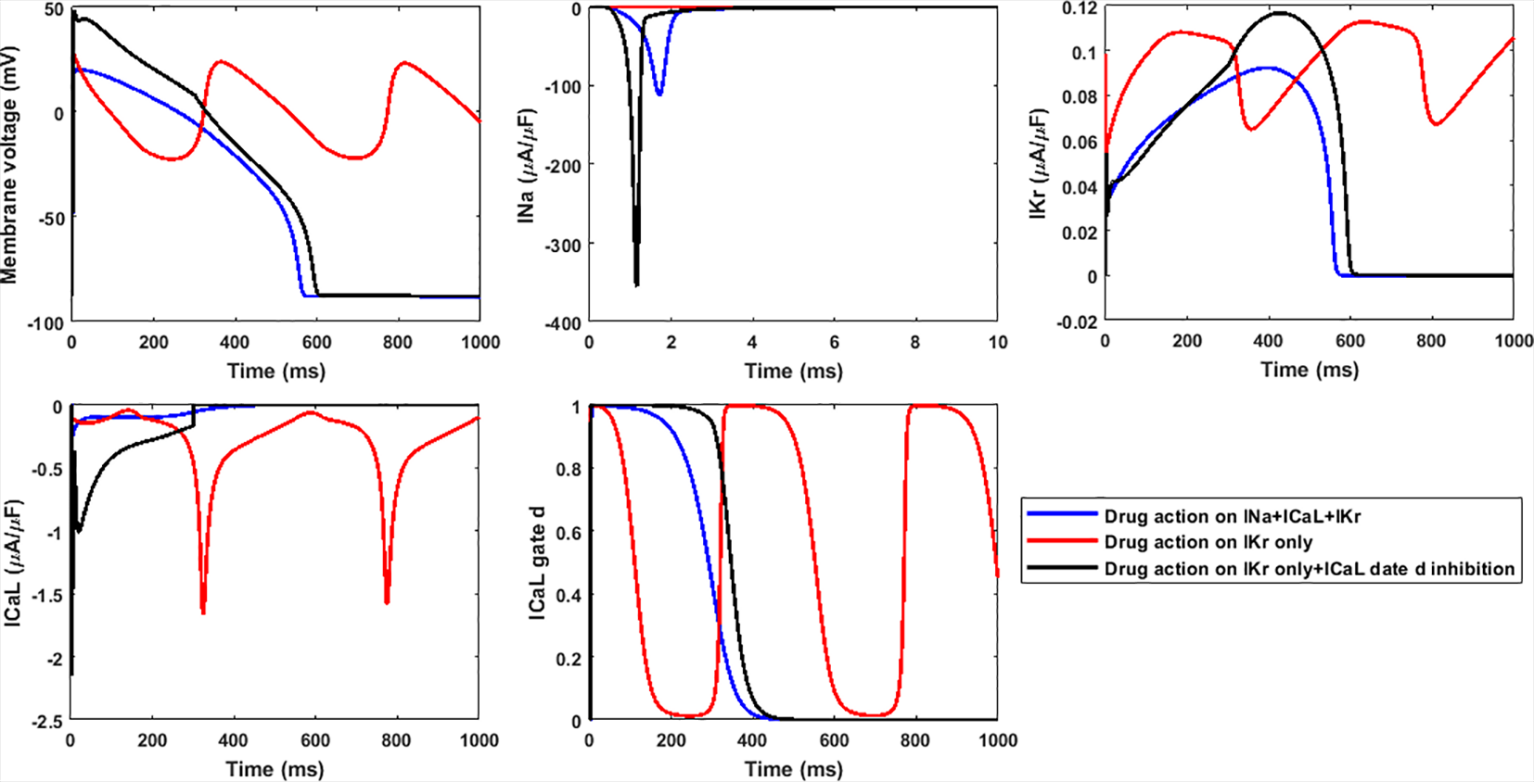
The lack of inhibition to I_CaL_ re-activation by only considering hERG block leads to higher inducibility of repolarization abnormalities for 100×EFTPC_max_ Linezolid II. Gate *d* is the activation gate of I_CaL_.

The higher RA inducibility when considering hERG alone was mainly due to I_CaL_ re-activation under lower repolarization reserve. For example, when only hERG block was applied to Linezolid II, RFs were observed at 100×EFTPC_max_, with re-opening of the I_CaL_ activation gate (gate *d* in the ORd model) leading to oscillations in I_CaL_ and membrane potential (**Figure 7**, red traces). When hERG block alone was applied but the re-activation of I_CaL_ was inhibited (post upstroke inhibition of gate *d*), RFs were successfully suppressed (**Figure 7**, black traces). Since Linezolid II has a strong blockage effect on the L-type calcium channel, the consideration of all three channels yielded a much lower AP plateau. This further inhibited I_CaL_, preventing the occurrence of a positive feedback loop of I_CaL_ re-activation to trigger RAs (**Figure 7**, blue traces).

### Moderate Changes in IC_50_s Combined With Variable Hill Coefficients Are Relevant to Divergent Prediction Outcomes

**Figure 8:**
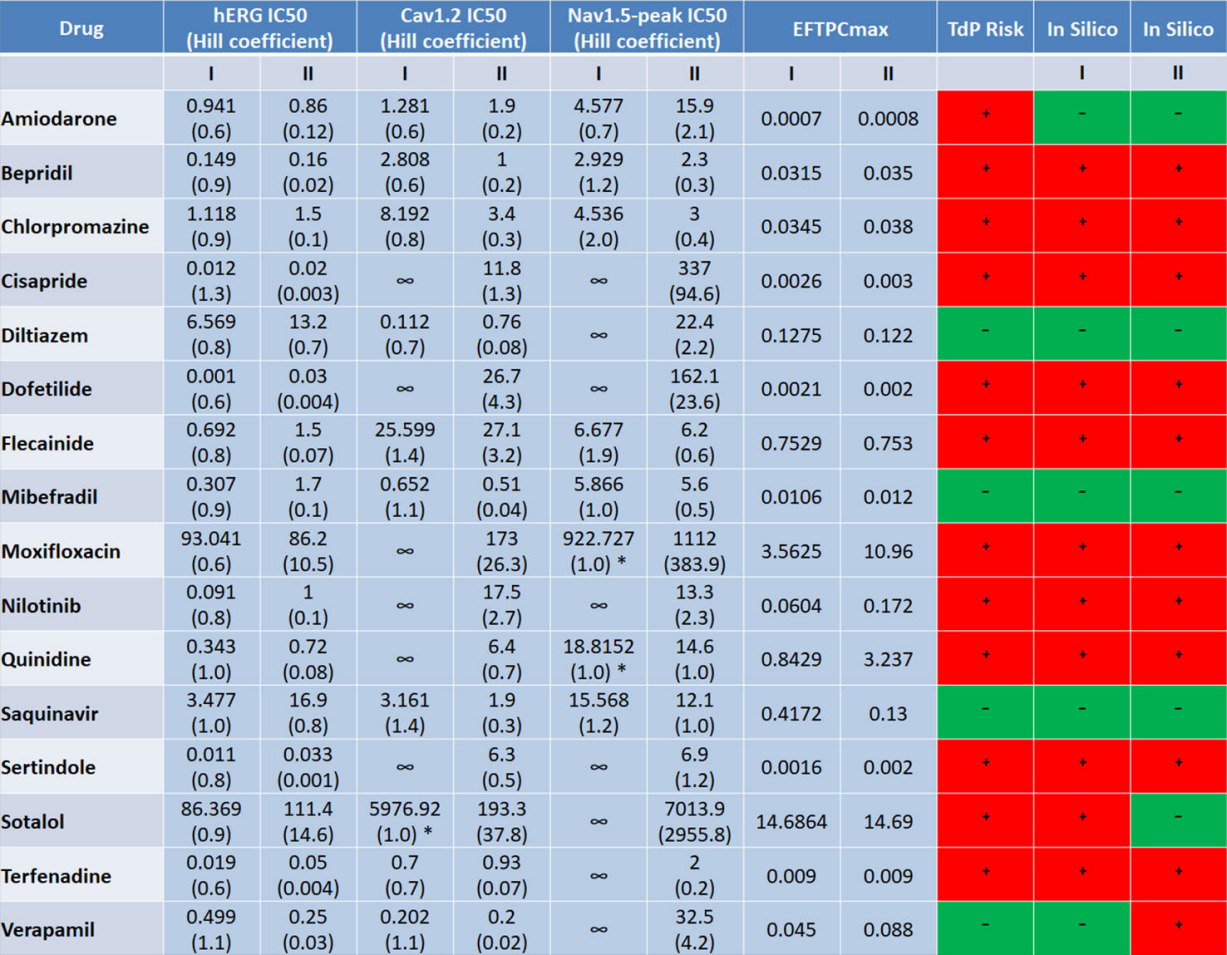
Comparison of the input ion channel IC_50_ values (μM), Hill coefficients, EFTPC_max_ (μM), and *in silico* prediction results for the 16 common compounds between the two datasets. * indicate the cases where IC_50s_ were estimated based on the percentage of ion channel blockage at the maximum tested concentration, with h equal to 1.

The ion channel information for Datasets I and II used in this *in silico* study came from different experimental groups, but they both contain information for the 16 compounds listed in **Figure 8**. In **Figure 8**, we listed the IC_50_ and Hill coefficient values of the three common ion channels between both datasets: hERG, Cav1.2 and Nav1.5-peak. For most compounds, the differences in ion channel IC_50_ datasets were moderate (<3-fold), while variations of Hill coefficient values were more significant between the two datasets. Despite the differences in IC_50_s, Hill coefficients and the number of affected ion channels, for 14 out of the 16 compounds, *in silico* predictions based on the two independent datasets are consistent: almost all correct, except for Amiodarone (**Figure 8**).

For Sotalol and Verapamil, *in silico* predictions based on the seven currents from Dataset I produced the correct outcome, while the predictions based on Dataset II lead to FP for Verapamil and FN for Sotalol. Applying only the blockages of hERG, Cav1.2 and Nav1.5-peak in Dataset I for Sotalol and Verapamil still produced correct classification results for both compounds (**Figure 6A**). This evidenced that it was not the number of ion channels but the different input values between the two datasets what led to the divergent prediction outcomes for these two drugs.

For Sotalol, the Cav1.2 IC_50_ value was much smaller with a steeper concentration-response curve (bigger Hill coefficient) in Dataset II. This corresponded to a more potent calcium channel blockage (100% blockage in Dataset II versus 20% blockage in Dataset I at 100x EFTPC_max_), providing the explanation for the FN prediction of Sotalol II. Verapamil II displayed very slow repolarization under 100×EFTPC_max_, with several models failing to reach resting membrane potential at the end of the simulation time (**Supplementary Figure S6A**). The difference in the prediction outcome of Verapamil between the two groups was due to the distinct input values in the two datasets: in Dataset II, Verapamil’s IC_50_ for hERG was about half the value that in Dataset I. In addition, the EFTPC_max_ concentration in Dataset II was almost doubled (**Figure 8**).

If the lower EFTPC_max_ in Dataset I (0.045 μM) was applied to the ionic profile of Verapamil II, RFs still occurred at 100×EFTPC_max_ (**Supplementary Figure S6B**), which proved the role of IC_50_s and Hill coefficients underlying the FP prediction. Therefore, for drugs with a multi-channel effect, moderate IC_50_ variations (<3-fold) combined with variable Hill coefficients can affect the accuracy of prediction outcomes.

## Discussion

In this study, we blindly performed 85 *in silico* drug trials for a total of 69 compounds based on two independent ion channel datasets with 16 overlapping drugs, using a computationally efficient population of human ventricular cell models. The main findings are the following:

1. For both datasets, the overall performance of the prediction was strong, with respective maximum accuracies of 83% and 80% for Dataset I and Dataset II.
2. When considering RAs for TdP prediction, decreasing the maximum testing concentration led to lower sensitivity without significant improvement of specificity, resulting in an optimal testing concentration of 100×EFTPC_max_.
3. I_CaL_ re-activation under reduced repolarization reserve caused by hERG block was the key mechanism underlying RAs. Calcium channel block decreased the propensity to RA. Therefore, only using hERG data decreased the specificity of predictions, and the optimal number of ion channels to achieve sufficient prediction accuracy was three: Nav1.5 (peak only), Cav1.2, and hERG.
4. For compounds with multiple ion channel potencies, moderate variations (<3-fold) in IC_50_ input values combined with variable Hill coefficients can lead to divergent prediction results.

### An Optimized Population of Human ORd-Based Models as an Efficient Tool for *In Silico* Risk Prediction and Mechanism Analysis

In this study, we used an optimized population of 107 models for blinded *in silico* drug trials, and we achieved accuracies of 83% for Dataset I and 80% for Dataset II if visual examination of the AP traces was performed. We showed that by using a small population of models that is more susceptible for RAs with uneven variations of the ionic currents, similar prediction accuracy can be achieved as a large population with even ionic current variations. Our results showed that this optimized population of models achieved a slightly lower accuracy than the previous population of 1,213 human ventricular models (Passini et al., 2017), but the computing efficiency was improved by 90% due to much smaller population size. Therefore, designing the optimized population of models is proved to be an efficient strategy to perform TdP risk prediction and physiological analysis (Passini et al., 2019).

We used the simple pore block model in this study, because it only required conventional measurements such as IC_50_ values and Hill coefficients. Other modeling studies have used more complex representations of ion channel kinetics under drug actions, such as Markov representations of the sodium (Morotti et al., 2014; Yang et al., 2016) or the hERG channels (Romero et al., 2015; Li et al., 2017) to incorporate the drug binding states. However, in order to gain sufficient experimental data to achieve accurate representations for these more detailed drug binding models, more specialized experimental protocols and settings are normally needed, which can be a speed limiting step for efficient screening of new compounds. The simple pore block model, on the other hand, enables a more efficient experimental data collection and potentially a faster application in new drug screening.

Another advantage of using populations of models with electrophysiological variability is that it enables direct observations of RAs after applying drug actions, which can be used to provide physiological insights. Although logistic regression models or machine learning algorithms can also achieve good performances in TdP risk classifications based on the ratios of IC_50_ and EFTPC (Kramer et al., 2013), or features of AP and intracellular calcium transients (Lancaster and Sobie, 2016), some of these algorithms do not enable direct observations of RAs, which compromises the mechanistic explorations for TdP risk. Combining machine learning algorithms and modeling could also be a useful new strategy (Parikh et al., 2017).

### Factors Affecting the Accuracy of Blinded *In Silico* TdP Risk Predictions

Although this blinded drug analysis using a small population of models achieved good accuracy for both groups of compounds, examination of some wrong predictions showed that additional information can be crucial for prediction outcome. For example, the predictions for Amiodarone were FN for both datasets in this study. However, if considering the possible lower plasma protein binding reported in literature (Lalloz et al., 1984; Latini et al., 1984), then testing at higher concentrations led to RAs occurrence with Amiodarone (Passini et al., 2017). Similarly, when taking into account the effect of Paliperidone, which is the major active metabolite of Risperidone, Passini et al. produced correct TdP risk classifications for Risperidone (Passini et al., 2017), since Paliperidone plays a more important role in QT prolongation (Suzuki et al., 2012). The information on drug metabolism was not optimized in this study because all compounds were blinded during the simulation process. A recent analysis by (Leishman, 2019) highlights the critical need to address contributions from clinically relevant metabolites in the qualification process to assure that the predictive performance of a new in silico model can address the pro-arrhythmic risk of exposure to both the parent drug and metabolites.

Another factor that affected the prediction accuracy was the definition of RAs. Based on our current definition, three compounds in Dataset II (Ceftriazone II, Linezolid II and Phenytoin II) were misclassified as RAs by the automated algorithm due to their very weak upstrokes and late peaks at the highest testing concentration (**Supplementary Material Figure S5**). Correcting such misclassifications led to a higher accuracy of 80% for Dataset II. This change in prediction revealed that, on one hand, standardized criteria should be proposed for the definition of RAs, especially if models are to be used for high-throughput drug screening; on the other hand, there are exceptional AP morphologies produced by high dose drugs that may require manual inspections and alternative explanations.

In addition, the accurate classifications of TdP-positive or TdP-negative drugs are benchmarks that are crucial in assessing the performance of *in-silico* modeling. Due to the controversial classification of TdP risk for some drugs, the performance of *in-silico* modeling would be affected. Finally, the inputs, i.e., ion channel potencies determined experimentally, and EFTPC_max_ obtained clinically, determine the accuracy of TdP prediction.

### TdP Scores Computed Up to 30 Folds of EFTPC_max_ Are Not Sufficient for the *In Silico* Predictions of Conditional TdP Risk

In order to incorporate the effects of inter-subject variability in drug binding and metabolism rates, as well as the uneven intra-subject drug distributions in the body, drug overdose is often used in both *in vitro* animal experimental tests and *in silico* simulations. In rabbit isolated Langendorff hearts, 30×EFTPC_max_ was shown to be sufficient without incurring TdP risk of potentially beneficial drugs (Lawrence et al., 2006). In this study, we also explored the effect of maximum testing concentration on the accuracy of predictions. We found that decreasing the testing concentration to 30×EFTPC_max_ can only improve specificity of predictions for Dataset II, but at the same time, the lower maximum testing concentration led to more FN predictions. For both datasets, the overall accuracy was lower under 30×EFTPC_max._ Similarly, the previous study using 1213 models also showed that the optimal maximum testing concentration is 100×EFTPC_max_ for the best accuracy (Passini et al., 2017).

We also noted that some FN predictions in this study (Clozapine II, Paroxetine II, Voriconazole II, and also Saquinavir II, Dasatinib II if considering CredibleMeds^®^ classifications), were also FN in the previous 1,213 population of models. Interestingly, although classified as risky, these FN compounds were considered to have possible or conditional TdP risk under the latest classification of CredibleMeds^®^. Cilostazol II (and Donepezil II by CredibleMeds^®^ classification) only induced EAD in one model under 100×EFTPC_max_ in the previous population, corresponding to the lowest TdP scores in the risky category (Passini et al., 2017). Therefore, for compounds with possible or conditional TdP risk, more detailed investigations need to be performed to take into account other factors in addition to the overdose, such as existing disease conditions, drug interaction, and metabolites. In our recently published paper, we reported the electromechanical window as a sensitive biomarker to improve the prediction of TdP risk for 40 reference compounds under lower tested concentrations (Passini et al., 2019), and future studies can test the prediction accuracy of combining electromechanical window for compounds with conditional TdP risk.

### *In Silico* Drug Trials Based on Nav1.5, Cav1.2, and hERG Generate Robust Prediction Results Without Compromising Efficiency

Previous experiments conducted in isolated ventricular myocytes or Langendorff-perfused animal hearts showed compounds with sodium or calcium blockage effects such as Lidocaine, Ranolazine, Nifedipine and Verapamil, can suppress EAD and prevent hERG blocker-induced TdP (Abrahamsson et al., 1996; Milberg et al., 2005; Yamada et al., 2008; Farkas et al., 2009; Milberg et al., 2012; Parikh et al., 2012). Therefore, it is essential to extend the TdP risk prediction from hERG-based analysis to a multiple-channel assay, which is the principle underlying this study and the CiPA initiative (Sager et al., 2014; Colatsky et al., 2016). In this study, we compared the effects of simulating only hERG blockage against simulating multiple ion channel blockages. For Dataset I, where the analysis was based on seven ion channel data from (Crumb et al., 2016), peak Nav1.5, Cav1.2, and hERG were the minimum set of ion channels with best efficiency for predictions, while for specific drugs, which have strong potency on other ion channels, predictions could improve by including these additional effects in simulations. As for the Dataset II prediction, based on data from (Kramer et al., 2013), only considering hERG significantly decreased specificity, and although sensitivity was slightly improved, the overall accuracy was also compromised. This is consistent with the previous hypothesis underlying CiPA, i.e., that simulating multiple channel blockages achieve more accurate predictions than only considering hERG (Gintant, 2011).

By using human ventricular cell models of electrophysiology, we were able to provide mechanistic explanations of the increased inducibility of RAs under hERG block. Our results showed that I_CaL_ re-activation was the key mechanism of RAs under hERG block, which was consistent with the mechanism revealed by sheep Purkinje fiber experiments (January et al., 1988; January and Riddle, 1989) and previous modeling investigations (Zeng and Rudy, 1995; Passini et al., 2016). Therefore, if calcium block effect is not considered, the TdP risk of a compound may be overestimated.

### Moderate Variations in IC_50_s Combined With Variable Hill Coefficients Affect *In Silico* Prediction Accuracy

IC_50_s as well as the steepness of the concentration-response curve of a same drug can vary across experiments and datasets (Kirsch et al., 2004; Yao et al., 2005; Milnes et al., 2010; Fermini et al., 2016; Passini et al., 2017). In this study, we also aimed to explore the effect of IC_50_ and Hill coefficient inputs on the stability of *in silico* predictions. By comparing the simulation results of 16 common drugs, 14 drugs showed consistent results across datasets. Considering the same ion channels (Nav1.5-peak, Cav1.2, and hERG), the overall accuracy for the 16 common drugs was slightly higher using the Crumb’s input values (14 correct) than the Kramer’s values (13 correct). This difference could originate from the experimental measurements: 1) the patch clamp experiments in the Crumb’s dataset was performed manually and mostly at physiological temperature, while in Kramer’s dataset, the experiments were conducted using automated patch clamp at ambient temperature; 2) for hERG and Cav1.2, Crumb’s dataset used AP waveform voltage protocols, while Kramer’s data were generated using step protocols (Kramer et al., 2013; Crumb et al., 2016). These differences in experimental settings may have contributed to the variability in IC_50_ and Hill coefficient measurements, and some variations in key ionic currents may lead to divergent simulation outcomes. For instance, the low IC_50_ value and steep concentration-response curve of Cav1.2 explained the FN prediction of Sotalol II.

### From EADs to Clinical TdP Risk

In clinical settings, patients with structural heart disease and electrophysiological remodeling are at highest risk for drug induced arrhythmia. Experimental work showed that cardiomyocytes isolated from structural heart disease patients with a history of ventricular tachycardia were significantly more prone to the development of EADs (Coppini et al., 2013), and EADs were frequently observed in whole heart experimental recordings of aged or diseased animals, as well as in human whole ventricle simulations (Morita et al., 2009; Milberg et al., 2012; Dutta et al., 2016; Van Nieuwenhuyse et al., 2017). In this study we considered an optimized population of models with low repolarization reserve, including weak I_Kr_, I_Ks_, and I_NaK_, together with strong I_NaL_, I_CaL_, and I_NaCa_, which are observed in multiple diseases, such as long QT syndrome (Schwartz Peter et al., 2012), hypertrophic cardiomyopathy (Coppini et al., 2013), and heart failure (Shamraj et al., 1993; Ambrosi et al., 2013). Therefore, the optimized population of models was designed to favor the generation of EAD and RF, but also to include possible electrophysiological remodeling occurring in patients at higher risk of developing drug-induced arrhythmias.

EADs have been frequently observed in single cells as well as in whole-heart and tissue experimental recordings and simulations in human and animal hearts (Sato et al., 1993; Morita et al., 2009; Sato et al., 2009; Milberg et al., 2012; Coppini et al., 2013; Dutta et al., 2016; Van Nieuwenhuyse et al., 2017). Additional pro-arrhythmic mechanisms such as increased dispersion of repolarization can also provide the substrate for the development of reentrant arrhythmia, and drugs with hERG blockage effects can amplify the intrinsic spatial dispersion of repolarization (Antzelevitch, 2005; Dutta et al., 2016). For instance, low therapeutic concentrations of quinidine preferentially prolonged APD in the midmyocardial cells (Antzelevitch et al., 1999), creating a vulnerable condition across the ventricular wall. In addition, cellular coupling has important roles in modulating EAD generations at tissue level (Pueyo et al., 2011). Although electrotonic coupling can smooth the chaotic EAD behavior (Weiss et al., 2010), regional EADs can propagate into the heterogeneous substrates, resulting in reentry and TdP patterns (Dutta et al., 2016; Vandersickel et al., 2016).

One effective approach to include the tissue effects in TdP risk predictions is whole ventricle simulations (Sato et al., 2009; Moreno et al., 2011; Trayanova, 2011; Dutta et al., 2016; Vandersickel et al., 2016; Martinez-Navarro et al., 2019), however they are computationally much more expensive than the current cell model population which has already shown strong performance. In this study we did not aim to equate EADs and TdP, but rather to use EADs as a pro-arrhythmic risk marker that is mechanistically linked to TdP. Given that the predictive accuracy of populations of models is high, computationally expensive simulations are not necessary. However, whole-ventricular simulations are very valuable for investigating mechanisms of arrhythmia as shown by (Sato et al., 2009; Moreno et al., 2011; Trayanova, 2011; Dutta et al., 2016; Vandersickel et al., 2016; Martinez-Navarro et al., 2019). Whole heart electrophysiology is also complicated by heart rate changes, which are regulated by the autonomic nervous system and hormones. For example Isoproterenol, a β-adrenergic receptor agonist, was used to terminate TdP by increasing heart rate and decreasing the dispersion of repolarization (Surawicz, 1989). Future studies could be performed by evaluating an agent’s TdP risk under β-adrenergic stimulations (Heijman et al., 2011; Tomek et al., 2017; Tomek et al., 2019).

## Conclusion

Through this blinded *in silico* drug trial, we demonstrated that computer simulations utilizing optimized population of human ventricular cell models are useful tools for high-throughput TdP risk predictions, and the minimum set of ion channels required for reliable predictions with highest computational efficiency are Nav1.5 (peak), Cav1.2, and hERG. For drugs with a multi-channel effect, moderate IC_50_ variations (<3-fold) combined with variable Hill coefficients could affect the accuracy of *in silico* predictions.

## Data Availability Statement

The datasets generated for this study are available on request to the corresponding authors.

## Author Contributions

All the authors conceived and designed the study. XZ performed the in silico drug assays, analyzed simulation results, and drafted the manuscript. YQ provided blinded drug potency datasets and performed drug classifications. XZ and YQ prepared the figures. YL tested the software. XZ, YQ, EP, AB-O, YL, HV and BR interpreted the results. All the authors edited and revised the manuscript.

## Funding

This study was funded by Amgen Inc. This work was supported by a Wellcome Trust Fellowship in Basic Biomedical Sciences to BR (100246/Z/12/Z and 214290/Z/18/Z) and a British Heart Foundation (BHF) Intermediate Basic Science Fellowship to AB-O (FS/17/22/32644). The authors also acknowledge additional support from the CompBioMed Centre of Excellence in Computational Biomedicine (European Commission Horizon 2020 research and innovation programme, grant agreement No. 675451), an NC3Rs Infrastructure for Impart Award (NC/P001076/1), and the Oxford BHF Centre of Research Excellence (RE/13/1/30181) and the TransQST project (Innovative Medicines Initiative 2 Joint Undertaking under grant agreement No 116030, receiving support from the European Union’s Horizon 2020 research and innovation programme and EFPIA). This work made use of the facilities of the UK National Supercomputing Service (Archer Leadership Award e462, Archer RAP Award 00180) and a PRACE project (2017174226).

## Conflict of Interest

Authors YQ, YL, and HV were employed by company Amgen Inc.

The remaining authors declare that the research was conducted in the absence of any commercial or financial relationships that could be construed as a potential conflict of interest.

The authors declare that this study received funding from Amgen Inc. The funder had the following involvement with the study: providing the blinded drug potency datasets for the simulations, and evaluating the accuracy of the blinded drug trial.

## Abbreviations

AP, action potential; APD, action potential duration; CiPA, Comprehensive in Vitro Proarrhythmia Assay initiative; EAD, early afterdepolarization; EFTPC_max_, maximal effective free therapeutic concentration; FDA, Food and Drug Administration; IC_50_, the concentration for 50% ion channel inhibition; I_CaL_, the L-type calcium current; I_Na_, the fast sodium current; I_NaCa_, the sodium-calcium exchanger current; I_NaK_, the sodium potassium pump current; I_NaL_, the late sodium current; I_K1_, the inward rectifier potassium current; I_Kr_, the hERG current, also known as the rapid delayed rectifier potassium current; I_Ks_, the slow delayed rectifier potassium current; I_to_, the transient outward potassium current; ORd model, O’Hara-Rudy dynamic human ventricular action potential model; QTc, Q-T interval on electrocardiogram corrected with heart rate; RA, repolarization abnormality; RF, repolarization failure; TdP, Torsades de Pointes; TP, true positive; TN, true negative; FP, false positive; FN, false negative; PPV, positive predictive value; NPV, negative predictive value.
