# Supplementary material for: Blinded In Silico Drug Trial Reveals the Minimum Set of Ion Channels for Torsades de Pointes Risk Assessment
Source: Front Pharmacol. 2020 Jan 30;10:1643. doi: 10.3389/fphar.2019.01643 (PMC7003137; doi:10.3389/fphar.2019.01643)
Supplement: Supplementary file 1 [file DataSheet_1.docx]

Supplementary Material

# Generation of the optimized population of human ventricular cell models

Based on the O’Hara-Rudy dynamic model, a computationally efficient population of 107 human ventricular action potential (AP) models, all of them in agreement with healthy human experimental recordings (Britton et al., 2017), was developed for the assessment of repolarization abnormalities (RA) under drug action (Passini et al., 2019). The population was designed based on established knowledge on ionic profiles susceptible to RAs (Passini et al., 2016). In this optimized population, the conductances for the rapid delayed rectifier potassium current (G_Kr_), the slow delayed rectifier potassium current (G_Ks_) and the sodium potassium pump (G_NaK_) tended to be weaker than those of the baseline O’Hara-Rudy model, whereas the conductances of the late sodium current (G_NaL_), the L-type calcium current (G_CaL_) and the sodium-calcium exchanger (G_NaCa_) tended to be stronger as shown in Table S1. The full list of parameter scaling factors is in Table S5.

| **Model Parameter** | **Variation Range** |
| --- | --- |
| G_Na_ | 30%-200% |
| G_NaL_ | 100%-200% |
| G_to_ | 0%-200% |
| G_Kr_ | 45%-100% |
| G_Ks_ | 0%-100% |
| G_K1_ | 30%-200% |
| G_NaCa_ | 100%-200% |
| G_NaK_ | 30%-100% |
| G_CaL_ | 100%-200% |

Table S1: Parameter variation range in the optimized population of models: conductances of the fast sodium current (G_Na_), the late sodium current (G_NaL_), the transient outward potassium current (G_to_), the rapid delayed rectifier potassium current (G_Kr_), the slow delayed rectifier potassium current (G_Ks_), the inward rectifier potassium current (G_K1_), the sodium-calcium exchanger (G_NaCa_), the sodium potassium pump (G_NaK_) and the L-type calcium current (G_CaL_).

# Prediction results based on the classifications of CredibleMeds^®^ database


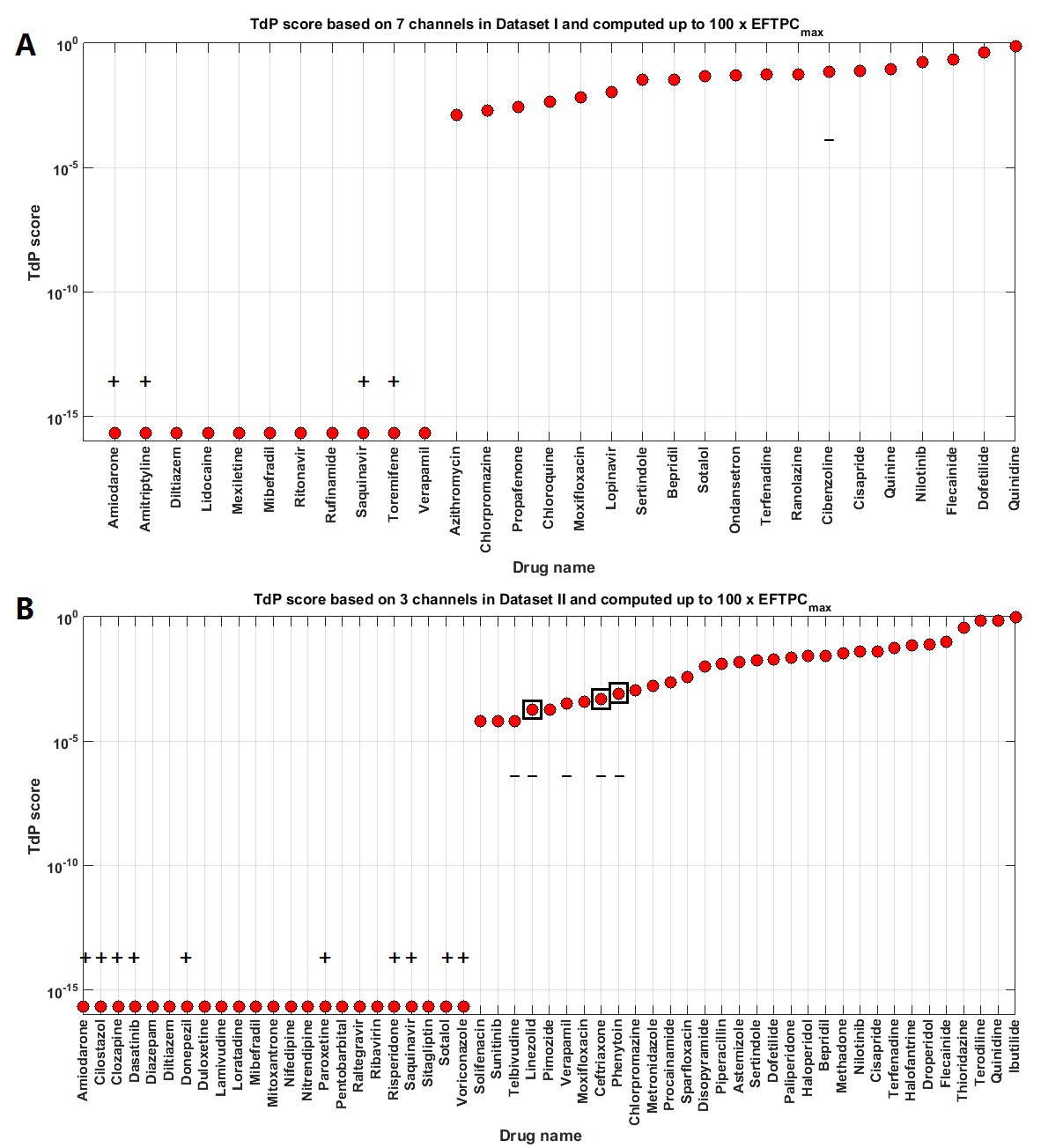


Figure S1: TdP risk assessment based on all the available ion channel inputs and up to 100 folds of EFTPC_max_ concentrations using the latest drug classification of CredibleMeds^®^. A) Dataset I TdP risk assessment based on all data from 7 ion channels (Crumb et al., 2016). B) Dataset II TdP risk assessment based on all information from 3 ion channels (Kramer et al., 2013). Classifications inconsistent with current clinical reports are labeled with black ‘**+**’ and ‘**–**’: ‘**+**’ implies the true classification should be risky, while ‘**–**’ means the true classification should be safe. Black squares in panel B highlight the compounds whose TdP risk was generated by the misclassification of the automated algorithm, and visual examinations of the simulated AP traces revealed they did not produce RAs.

|  | Dataset I | | Dataset II | | |
| --- | --- | --- | --- | --- | --- |
|  | Clinical TdP (+) | Clinical TdP(-) | Clinical TdP (+) | Clinical TdP(-) | |
| In silico (+) | 18 | 1 | 27 | 5 | **2** |
| In silico (-) | 4 | 7 | 10 | 13 | **16** |
| Sensitivity | 82% | | 73% | **73%** | |
| Specificity | 88% | | 72% | **89%** | |
| PPV | 95% | | 84% | **93%** | |
| NPV | 64% | | 57% | **62%** | |
| Accuracy | 83% | | 73% | **78%** | |

Table S2: Accuracy of the in silico TdP risk predictions using the 107 human ventricular cell population of models with maximum testing concentrations of 100 folds of EFTPC_max_ based on the latest TdP classification of CredibleMeds^®^. For Dataset II, bold scores indicate the true accuracy of the model predictions after fixing the misclassifications of the automated RA detection algorithm.


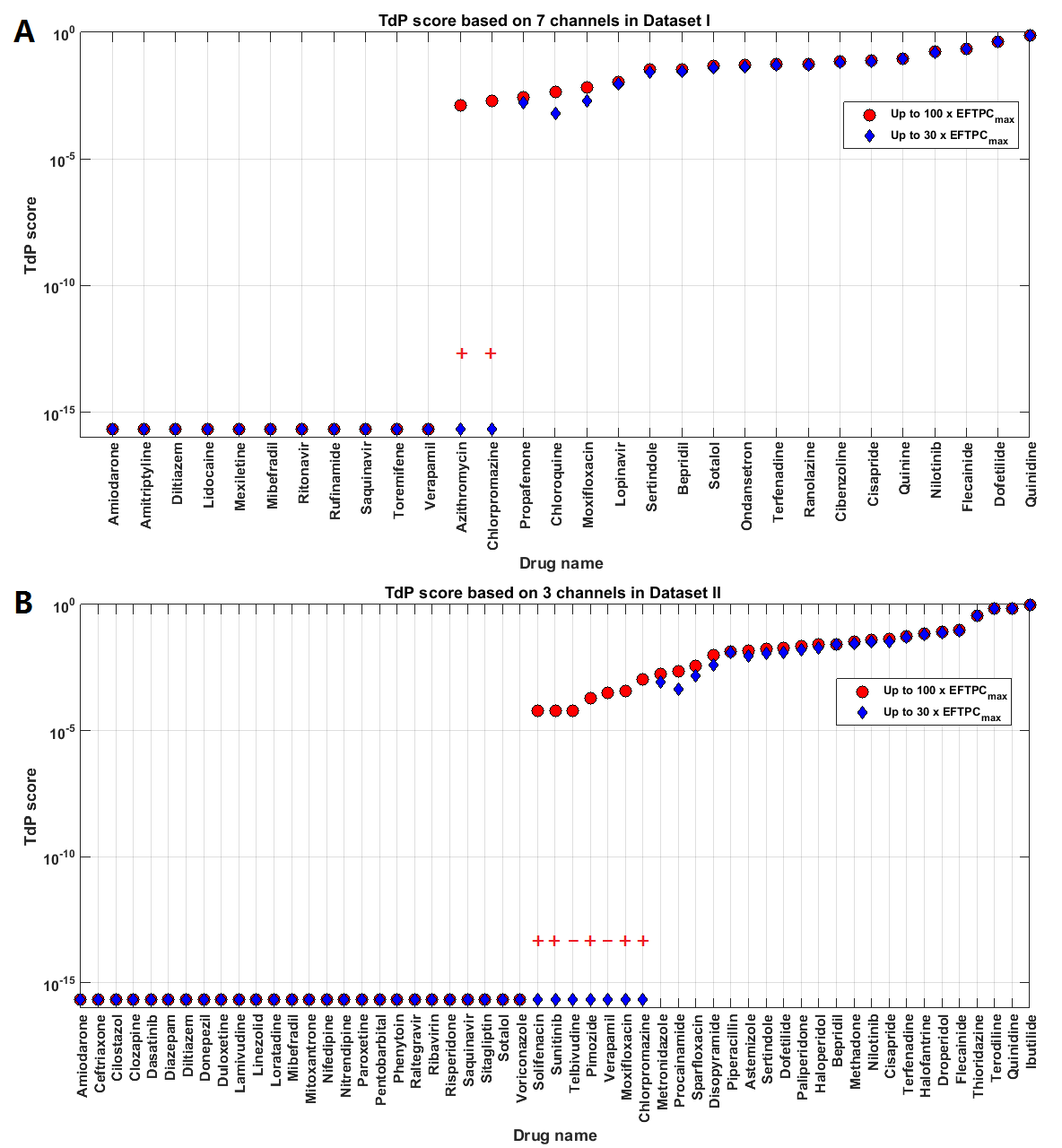


Figure S2: Comparison of TdP risk between up to 30-fold EFTPC_max_ and up to 100-fold EFTPC_max_ for Dataset I (A) and Dataset II (B) using the latest drug classification of CredibleMeds^®^. Red ‘**+**’ and ‘**–**’ show the classification changes caused by lower testing concentration: ‘**+**’ implies the true classification should be risky, while ‘**–**’ means the true classification should be safe. The three Dataset II compounds whose TdP risk was misclassified by the automated algorithm were corrected in this figure.

|  | Dataset I | | Dataset II | | |
| --- | --- | --- | --- | --- | --- |
|  | 100xEFTPC_max_ | 30x EFTPC_max_ | 100xEFTPC_max_ | | 30x EFTPC_max_ |
| Sensitivity | 82% | 73% | 73% | **73%** | 59% |
| Specificity | 88% | 88% | 72% | **89%** | 100% |
| PPV | 95% | 94% | 84% | **93%** | 100% |
| NPV | 64% | 54% | 57% | **62%** | 55% |
| Accuracy | 83% | 77% | 73% | **78%** | 73% |

Table S3: Comparisons of the in silico TdP risk predictions between maximum testing concentrations of 30 folds and 100 folds of EFTPC_max_ based on the latest TdP classification of CredibleMeds^®^. For Dataset II, bold scores indicate the true accuracy of the model predictions after fixing the misclassifications of the automated RA detection algorithm.


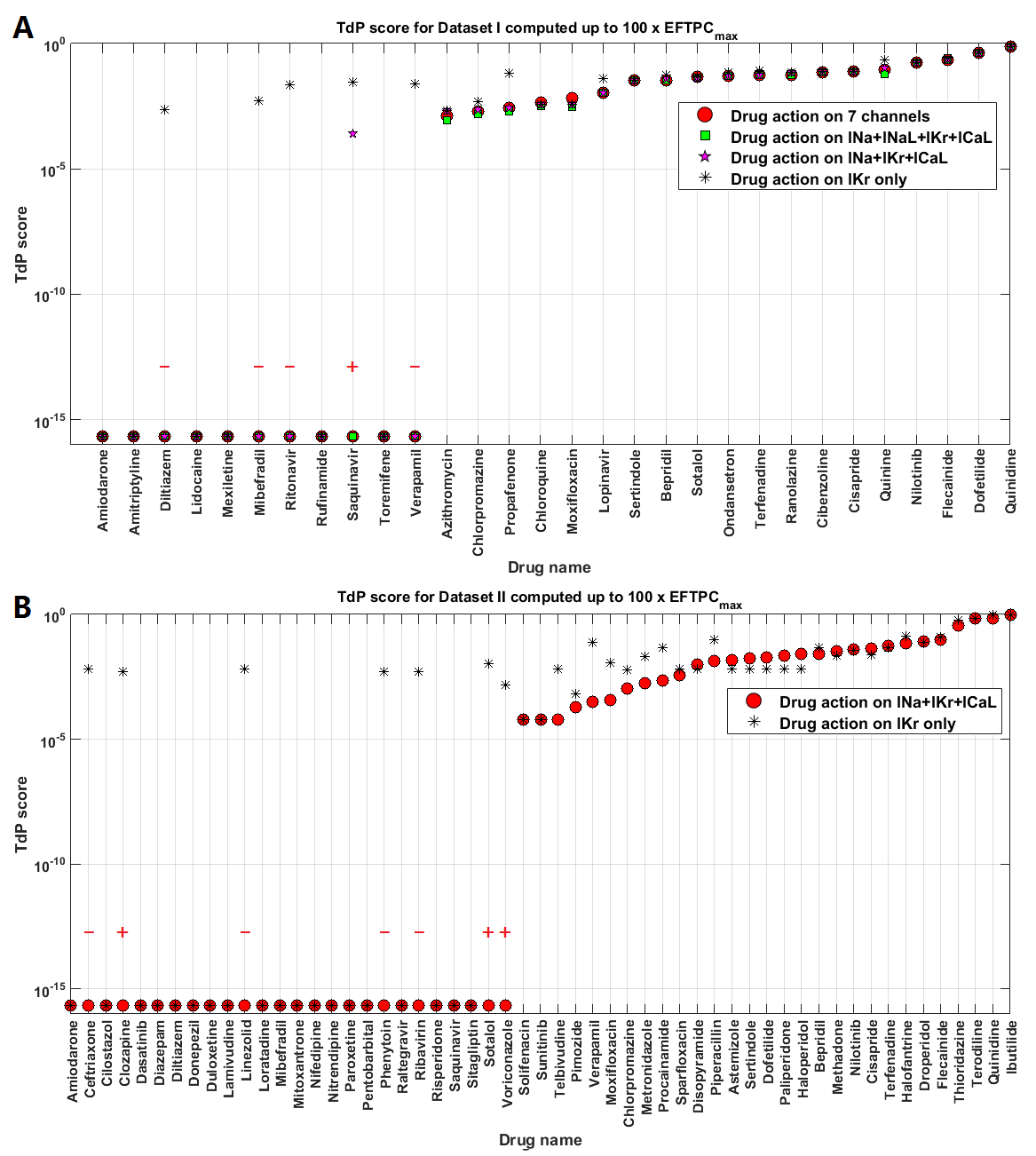


Figure S3: Comparison of TdP risk for different subsets of ion channel information for Dataset I (A) and Dataset II (B) using the latest drug classification of CredibleMeds^®^. Red ‘**+**’ and ‘**–**’ show the classification changes caused by different ion channel profiles: ‘**+**’ implies the true classification should be risky, while ‘**–**’ means the true classification should be safe. The three Dataset II compounds whose TdP risk was misclassified by the automated algorithm were corrected in this figure, and they did induce RAs under hERG only simulations.

|  | Dataset I | | | | Dataset II | | |
| --- | --- | --- | --- | --- | --- | --- | --- |
|  | 7 channels | 4 channels | 3 channels | 1 channel (hERG) | 3 channels | | 1 channel (hERG) |
| Sensitivity | 82% | 82% | 86% | 86% | 73% | **73%** | 81% |
| Specificity | 88% | 88% | 88% | 38% | 72% | **89%** | 67% |
| PPV | 95% | 95% | 95% | 79% | 84% | **93%** | 83% |
| NPV | 64% | 64% | 70% | 50% | 57% | **62%** | 63% |
| Accuracy | 83% | 83% | 87% | 73% | 73% | **78%** | 76% |

Table S4: Comparisons of the in silico TdP risk predictions between different sets of ion channel profiles based on the latest TdP classification of CredibleMeds^®^. For Dataset II, bold scores indicate the true accuracy of the model predictions of 3 channels after fixing the misclassifications of the automated RA detection algorithm.


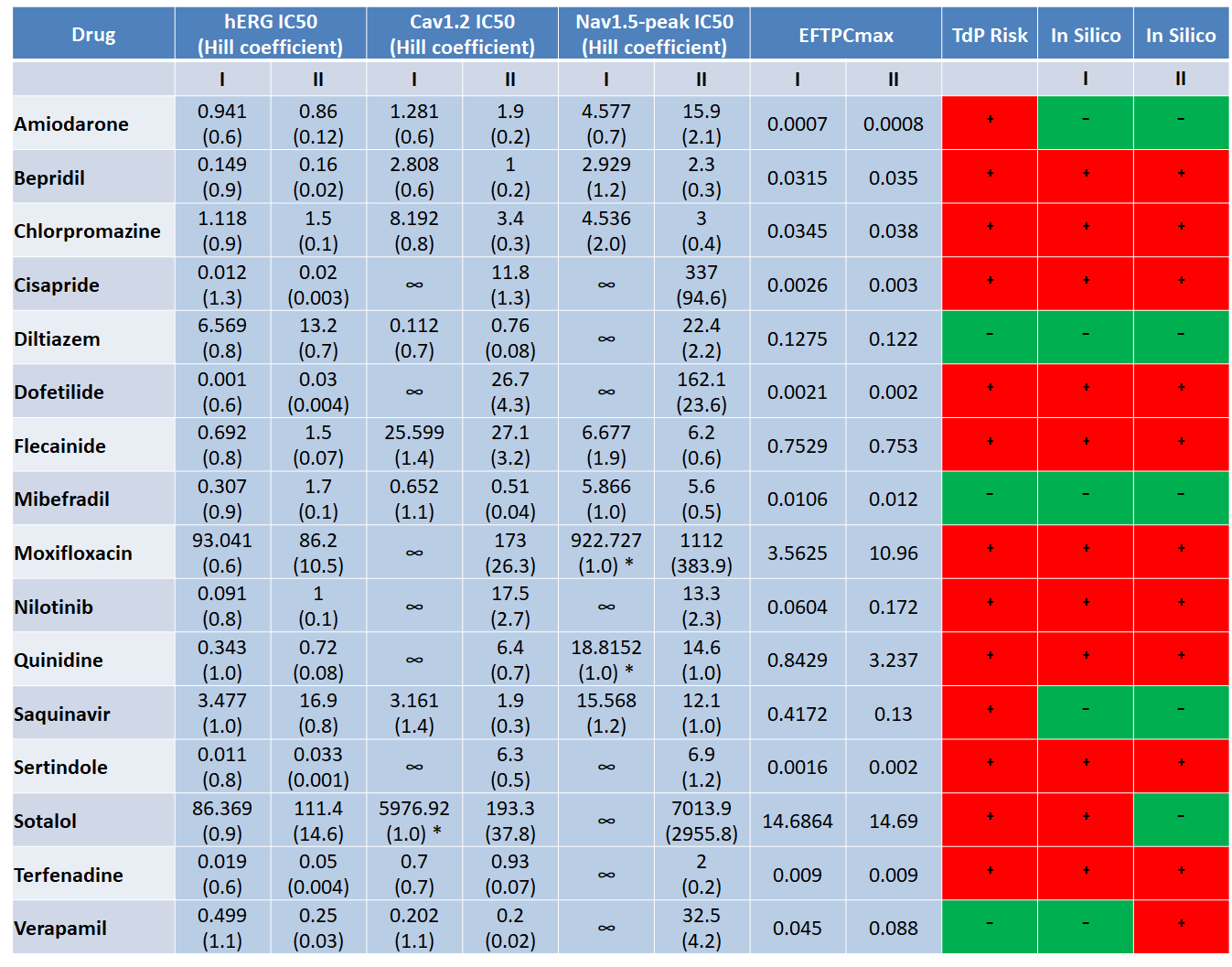


Figure S4: Comparison of the input ion channel IC_50_ values (μM), Hill coefficients, EFTPC_max_ (μM), and in silico prediction results for the 16 common compounds between the two datasets using the latest drug classification of CredibleMeds^®^. * indicate the cases where IC_50_ was estimated based on the percentage of ion channel blockage at the maximum tested concentration, with h equal to 1.

**Analysis of several false positive cases**


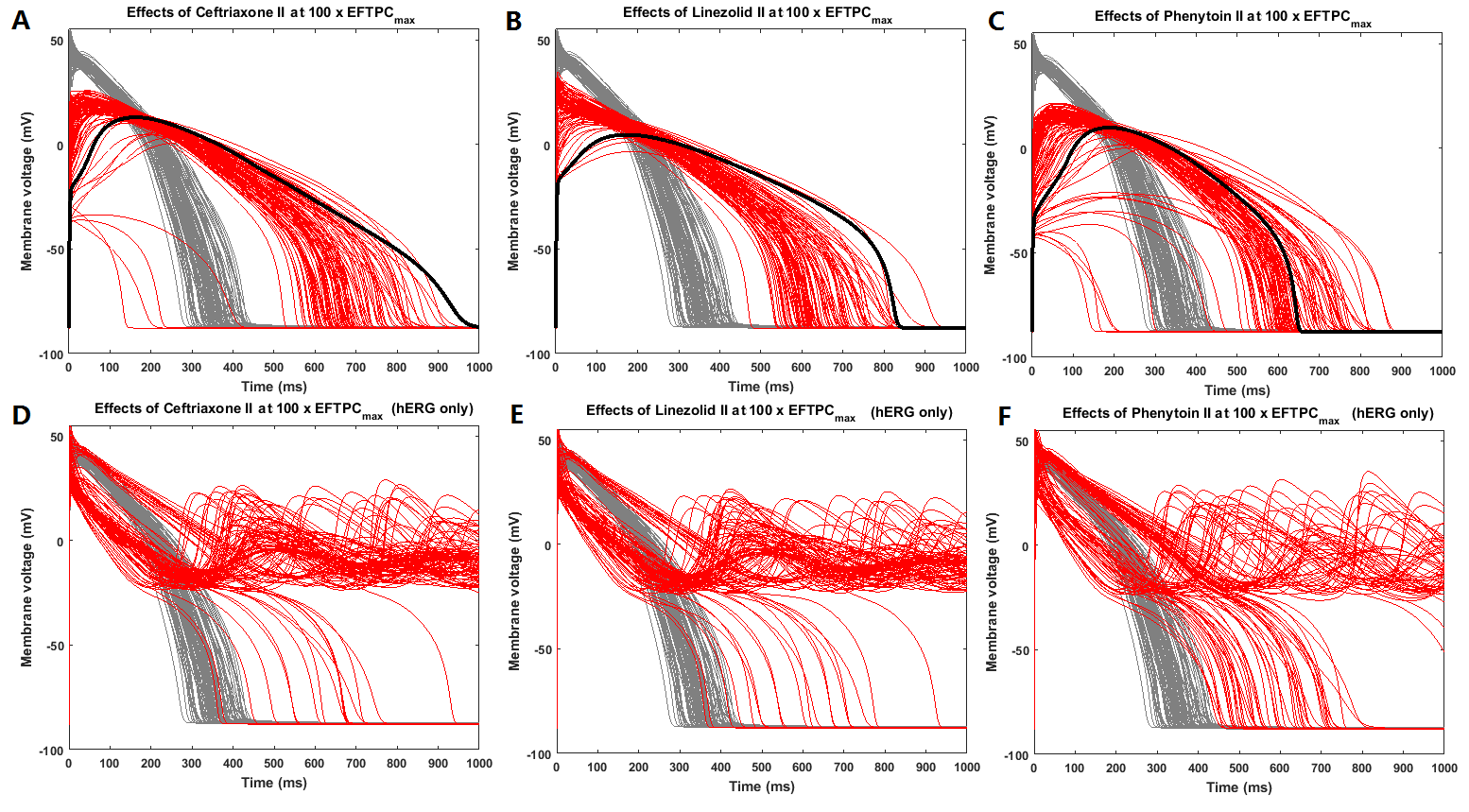


Figure S5: Effects of blocking 3 channels (A-C) versus blocking hERG only (D-F) at100-fold EFTPC_max_: A), D) Ceftriaxone II; B), E) Linezolid II; C), F) Phenytoin II. Grey: control condition; red: drug action; black: false positive under drug action. Bold black traces highlight the cases where the automated RA detection algorithm misclassified the traces as EADs due to their very late peak after 150 ms.


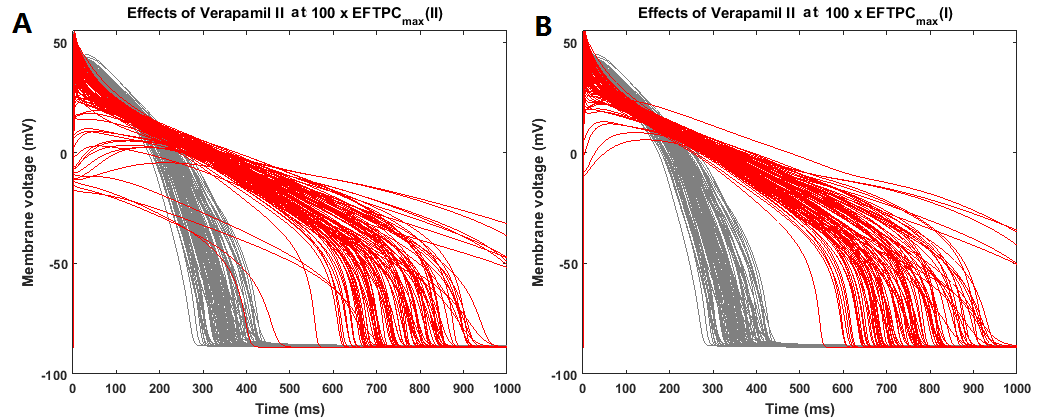


Figure S6: Effects of 100-fold EFTPC_max_ Verapamil II. A) at Kramer 100-fold EFTPC_max_; B) at Crumb 100-fold EFTPC_max_. Grey: control condition; red: drug action.

**Representative examples to demonstrate the effects of changing pacing frequency**


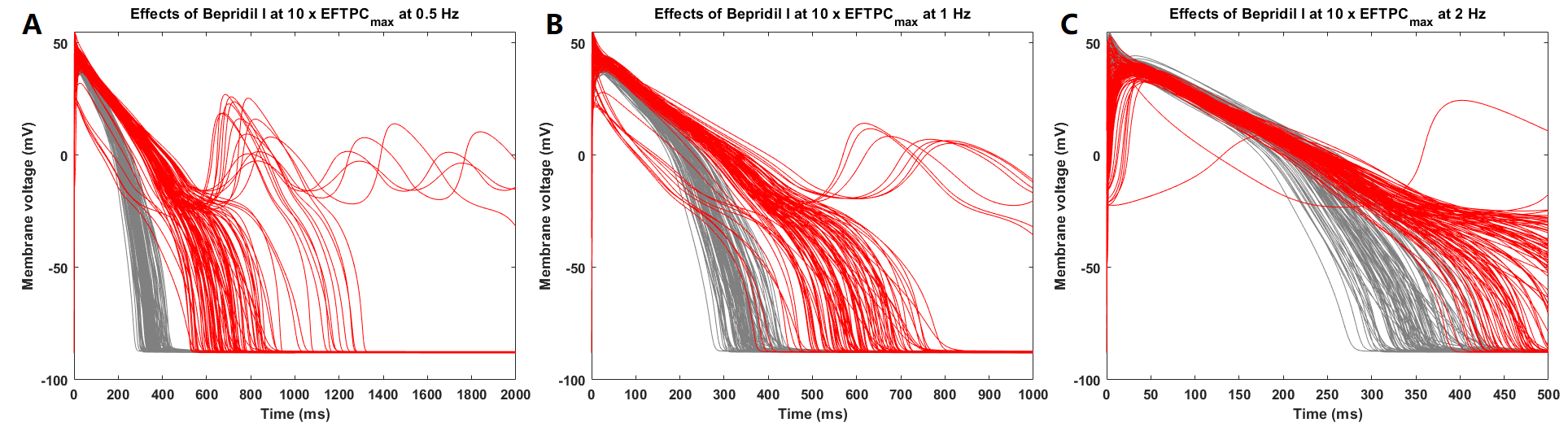


Figure S7: Effects of changing pacing frequency for a representative compound Bepridil I at 10-fold EFTPC_max_ at 0.5Hz (A), 1Hz (B) and 2Hz (C). Grey: control condition; red: drug action.


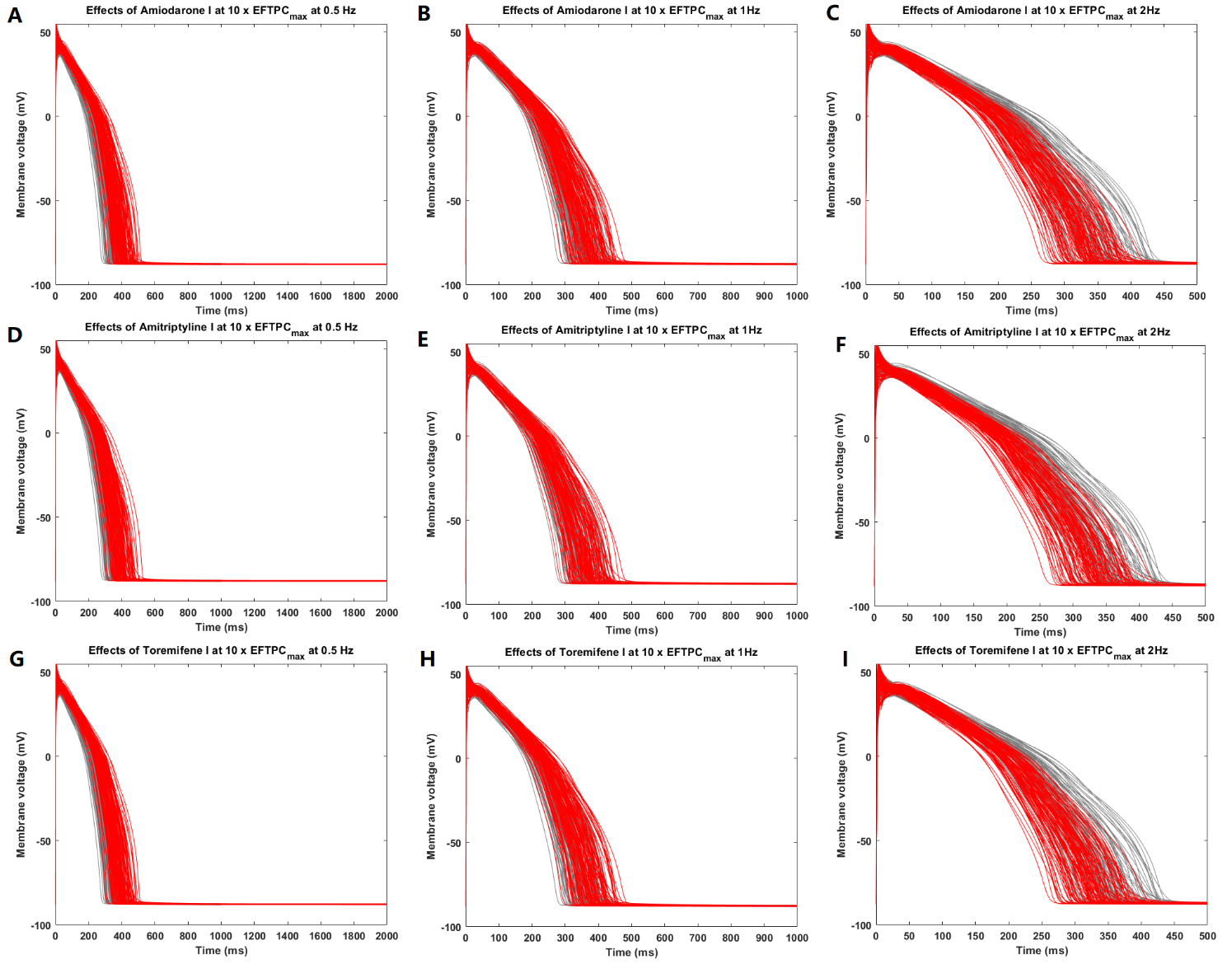


Figure S8: Effects of changing pacing frequency for representative false negative compounds at 10-fold EFTPC_max_. Amiodarone I at 0.5Hz (A), 1Hz (B) and 2Hz (C); Amitriptyline I at 0.5Hz (D), 1Hz (E) and 2Hz (F); Toremifene I at 0.5Hz (G), 1Hz (H) and 2Hz (I). Grey: control condition; red: drug action.


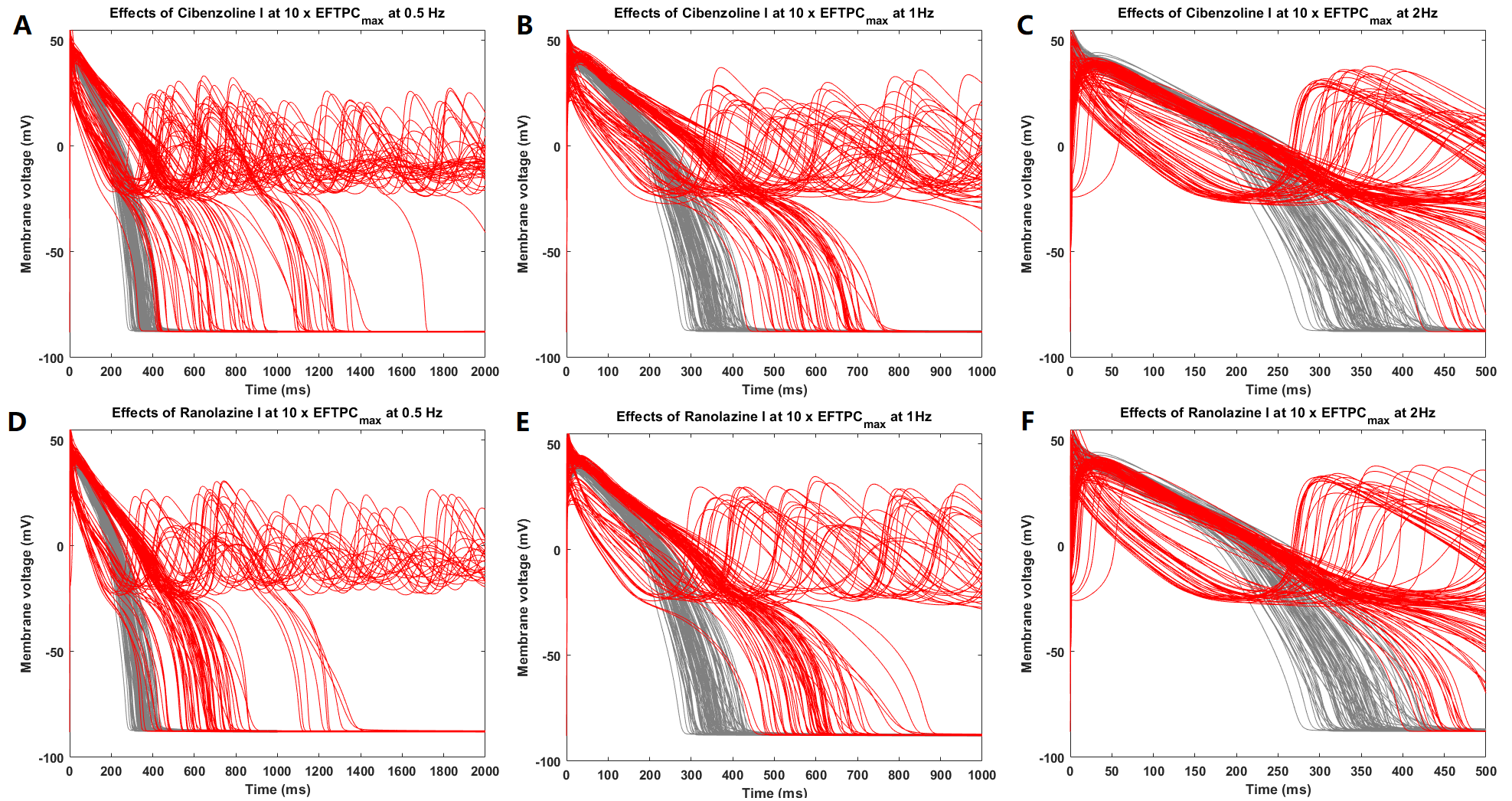


Figure S9: Effects of changing pacing frequency for representative false positive compounds at 10-fold EFTPC_max_. Cibenzoline I at 0.5Hz (A), 1Hz (B) and 2Hz (C); Ranolazine I at 0.5Hz (D), 1Hz (E) and 2Hz (F). Grey: control condition; red: drug action.

**References**

Britton, O. J., Bueno-Orovio, A., Virág, L., Varró, A., and Rodriguez, B. (2017). The Electrogenic Na(+)/K(+) Pump Is a Key Determinant of Repolarization Abnormality Susceptibility in Human Ventricular Cardiomyocytes: A Population-Based Simulation Study. *Front Physiol* 8, 278. doi:10.3389/fphys.2017.00278.

Crumb, W. J., Vicente, J., Johannesen, L., and Strauss, D. G. (2016). An evaluation of 30 clinical drugs against the comprehensive in vitro proarrhythmia assay (CiPA) proposed ion channel panel. *J Pharmacol Toxicol Methods* 81, 251–262. doi:10.1016/j.vascn.2016.03.009.

Kramer, J., Obejero-Paz, C. A., Myatt, G., Kuryshev, Y. A., Bruening-Wright, A., Verducci, J. S., et al. (2013). MICE models: superior to the HERG model in predicting Torsade de Pointes. *Sci Rep* 3, 2100. doi:10.1038/srep02100.

Passini, E., Mincholé, A., Coppini, R., Cerbai, E., Rodriguez, B., Severi, S., et al. (2016). Mechanisms of pro-arrhythmic abnormalities in ventricular repolarisation and anti-arrhythmic therapies in human hypertrophic cardiomyopathy. *Journal of Molecular and Cellular Cardiology* 96, 72–81. doi:10.1016/j.yjmcc.2015.09.003.

Passini, E., Trovato, C., Morissette, P., Sannajust, F., Bueno-Orovio, A., and Rodriguez, B. (2019). Drug-induced Shortening of the Electromechanical Window is an Effective Biomarker for in Silico Prediction of Clinical Risk of Arrhythmias. *Br. J. Pharmacol.* doi:10.1111/bph.14786.
